# The Action of Verbal and Non-verbal Communication in the Therapeutic Alliance Construction: A Mixed Methods Approach to Assess the Initial Interactions With Depressed Patients

**DOI:** 10.3389/fpsyg.2020.00234

**Published:** 2020-02-21

**Authors:** Luca Del Giacco, M. Teresa Anguera, Silvia Salcuni

**Affiliations:** ^1^Department of Social Psychology and Quantitative Psychology, University of Barcelona, Barcelona, Spain; ^2^Department of Developmental Psychology and Socialization, University of Padua, Padua, Italy; ^3^Faculty of Psychology, Institute of Neurosciences, University of Barcelona, Barcelona, Spain

**Keywords:** verbal and non-verbal communication, performative language, therapeutic alliance construction, mutual regulation, coordination processes, psychotherapy process, depression, mixed-methods approach

## Abstract

In psychodynamic psychotherapy, verbal (structures and intents) and non-verbal (voice and interruptions) dimensions of communication intertwine conveying information and determining the mutual regulation between therapist and patient through conversational sequences. The communication components interplay is the foundation for building the therapeutic alliance, a relational dimension that predicts a psychotherapy outcome and change, influenced by patient-therapist exchanges from the initial stages of their encounter. Depressed patients present specific verbal and non-verbal communication and show difficulties in developing and maintaining the therapeutic alliance. Based on the reviewed literature, the main aim of this study was to analyze how the action of specific communicative modes, implemented by the therapist and depressed patients, affect the reciprocal construction of the early therapeutic alliance by each participant during the mutual regulation processes. We employed a mixed methods approach based on a systematic observation of communication and alliance ruptures and repairs within the audio recordings and verbatim transcripts of 20 psychotherapy sessions (6,232 speaking turns) with seven depressed patients. The observational design was nomothetic, follow-up, and multidimensional. The choice of methodology is justified because we developed a comprehensive procedure that integrates an *ad hoc* indirect observation system (the Communicative Modes Analysis System in Psychotherapy), analyzing verbal and non-verbal communication, and an observational tool with deductive categories (the Collaborative Interactions Scale-Revised), assessing the therapeutic alliance construction. Once we confirmed the intra-and inter-observer reliability for the *ad hoc* system and the inter-rater reliability for the tool with deductive (or theoretical) categories, we performed descriptive statistics (to describe quantitatively communicative modes and alliance ruptures and repairs), lag sequential analysis (to detect stable patterns in communication-alliance interactions), and polar coordinate analysis (to identify significant relationships between communicative modes and alliance ruptures and repairs). Results confirm that the therapist's verbal (asking and exploring) and non-verbal (elaborating and cooperatively interrupting) modes and the depressed patients' verbal (asserting and exploring) and non-verbal (expressing emotions and cooperatively interrupting) modes determine stable patterns and significant associations with collaborative behaviors connected to the reciprocal construction of alliance by each participant. All this may provide professionals with useful information to increase the psychotherapy effectiveness with depressed patients.

## Introduction

According to the psychodynamic approach, the therapeutic setting is the place where the therapist and patient establish a specific and asymmetric dialogue to explore and co-construct meanings through the intertwinement of verbal and non-verbal communication (Molina et al., 2013).

In psychotherapy research, these components of communication have always been considered independent (Westland, 2015) and studied separately (e.g., Salvatore et al., 2010; Tomicic et al., 2011; Ruiz-Sancho et al., 2013). However, in recent decades, scholars have been acknowledging the mutual influence of verbal and non-verbal dimensions as interrelated phenomena that can occur sequentially and simultaneously during communicative exchanges (Jones and LeBaron, 2002; Westland, 2015).

Assuming that people “co-construct and negotiate meanings in their ongoing interactions” (Jones and LeBaron, 2002, p. 504), we developed an integrative model of communication in psychotherapy (Del Giacco et al., 2019) to overcome the limits of previous research, based on the notion of performative language from the Speech Act Theory (SAT; Searle, 2017). According to our model, verbal and non-verbal dimensions are linguistic acts expressing the intents of speakers who co-construct a dynamic relationship through a two-way process that oscillates between self-and mutual regulation and is connected to psychotherapy change (Martinez Guzman et al., 2014; Westland, 2015). Precisely, voice and interruptions, together with verbal communication, assume a fundamental role in co-constructing meanings as, from one hand, they provide information on the psychological messages and emotional states underlying the participants' behaviors and, on the other hand, they enrich the speech through their interaction even though they are separate components (Jones and LeBaron, 2002). Therefore, verbal communication (through the structural form and communicative intents of the content), voice (through prosodic modulations), and cooperative/competitive interruptions (through behaviors of involvement or dominance) interact by spreading information and determining the mutual regulation between participants in the form of conversational sequences, observable and recordable during communicative exchanges (Li, 2001; Valdés et al., 2010; Tomicic et al., 2015b; Westland, 2015).

Scholars (e.g., Adigwe and Okoro, 2016; Rocco et al., 2018) agree that the dynamic interaction of verbal and non-verbal components is the foundation for building a good therapeutic alliance (TA) (Martinez Guzman et al., 2014), a collaborative dimension whose quality depends on the mutual interaction between therapist and patient as well as their respective contributions (Lingiardi et al., 2016). Different authors have proven that the TA is an active agent in the process of change in psychotherapy (Colli and Lingiardi, 2009; Flückiger et al., 2018; Uckelstam et al., 2018; Vernmark et al., 2019). In particular, the TA in the initial stages of psychotherapy predicts a better outcome and change than the one measured in the middle of psychotherapy (Flückiger et al., 2018): it seems to be stronger in the first session with peaks during the third one (Ardito and Rabellino, 2011). This relational dimension consists of a continuous negotiation process between the patient's and therapist's needs and passes through rupture and repairs moments implemented by both participants that influence change (Safran et al., 2011; Locati et al., 2019). Precisely, ruptures manifest themselves through phases of lack of coordination characterized by non-cooperative behaviors between participants, while repairs through coordination phases identified by cooperative behaviors; both of them are expressed through verbal and non-verbal communication (Colli and Lingiardi, 2009; Morán et al., 2016; Colli et al., 2017). The therapist's capacity to acknowledge and manage these moments could lead the therapy to positive changes or negative results (Eubanks et al., 2018). Therefore, the intersubjective negotiation in the therapeutic relationship involves a reciprocal regulation process that can itself be a mechanism of therapeutic change (Safran and Muran, 2003, 2006; Martinez Guzman et al., 2014): shifts in the collaboration and coordination levels can be considered fundamental keys of change (Colli and Lingiardi, 2009; Lingiardi et al., 2016).

Even though the literature acknowledges that the TA manifests itself through verbal and non-verbal expressions (Morán et al., 2016), studies mainly focused on verbal interactions (e.g., Krause et al., 2016), giving little emphasis to research on non-verbal components (e.g., Rocco et al., 2018) and their interactions with the former (e.g., Martinez Guzman et al., 2014) in the TA construction. Therefore, deepening the relationship between communication and TA by considering the verbal and non-verbal dimensions as an integrated and interacting system (Del Giacco et al., 2019) may overcome the limits of the previous research and provide professionals with useful information to increase knowledge about building such a collaborative relationship and the therapy effectiveness.

Scholars attempted to determine what communicative actions patient and therapist reciprocally implement during change episodes, specific in-session segments characterized by verbal and non-verbal coordination between participants and associated with the TA co-construction (Mellado et al., 2017), confirming the essential role of verbal structures and intentions, voice, and interruptions. For example, Krause et al. (2016) detected that *asserting* something and *asking* for information represented the verbal structures connected to the coordination processes at the basis of change episodes and the TA construction. Furthermore, they proved that the patient tended to assert more than the therapist, while the latter was inclined to question more than the former. Dagnino et al. (2012) showed that *exploring* one's own or the other's experience was the main verbal communicative intention underlying the coordination sequences connected to change episodes in the initial stages of psychotherapy. Moreover, during this phase, patients tended to explore more than the therapist. Tomicic et al. (2015b) emphasized that, regardless of verbal content, both an *elaborative* and *emotional vocal quality* were associated with coordination processes between participants. Furthermore, the therapist highlighted a more elaborative voice than the patient, while the latter expressed a greater emotionality than the former in terms of vocal emission. Finally, Oka et al.^1^ confirmed the mediating role of interruptions in the TA construction, although the results showed little effect of the cooperative vs. the competitive type. However, the patient implemented more competitive interruptions than the therapist, while the latter tended to interrupt more cooperatively than the former. Since research on the relationship between interruptions and TA is scarce in psychotherapy, we relied on studies in the field of communication (e.g., Li et al., 2005; Cafaro et al., 2016) to assume that the *cooperative interruptions*^2^ can also support coordination processes in the TA construction.

Patients, therefore, live the therapeutic relationship and the alliance construction by manifesting different experiential and behavioral modalities through verbal and non-verbal communication (Tomicic et al., 2009; Valdés and Krause, 2015), as an expression of their psychological processes and symptoms (Valdés, 2014; Elvevåg et al., 2016). Depressed patients, in particular, show difficulties in developing and maintaining the TA because of the specific verbal and non-verbal correlates that define their communicative behaviors (Balsters et al., 2012; Smirnova et al., 2018). According to the psychodynamic approach, these behaviors reflect the broad range of depressed patients' defensive, adaptation, and cognition styles deriving from the early cognitive-affective representations where anger and aggression are predominant (Levy and Wasserman, 2009). This kind of patients has difficulty in accessing their inner world and emotions and in maintaining an adequate relational distance (Valdés, 2014; Valdés and Krause, 2015) which are manifested, on the one hand, through a rambling, repetitious, and vague speech (Bucci and Freedman, 1981), and from the other, through slow and monotonous speech with less volume and voice modulation (Rottenberg and Gotlib, 2004). These aspects vehicle the egocentric view of self, lack of empathy, interpersonal problems, and relational dependence typical of depressed patients who tend to exhibit hopelessness and passive-aggressive behaviors (Levy and Wasserman, 2009) through verbal and non-verbal communication that impact on the construction of a collaborative relationship.

As Hardy and Llewelyn (2015) point out, over the years, the study of the dynamics underlying the therapeutic relationship has involved the use of different methodologies (e.g., individual case studies, qualitative or quantitative analysis, naturalistic studies) and different analysis techniques (e.g., standardized methods, hermeneutics approaches, speech analysis) to provide empirical evidence aimed at explaining the role of factors that foster clinical change (e.g., Elliott et al., 2009; Eubanks et al., 2018; Smink et al., 2019). However, in recent decades, psychotherapy research has been moving toward an integrated approach of qualitative and quantitative methods, the mixed methods approach (Creswell and Plano Clark, 2017), to have a fuller picture of the ecological context of the therapeutic interaction supported by objective measures (Gelo et al., 2012; Bartholomew and Lockard, 2018). The systematic observation, deriving from this approach and considered being mixed methods in itself, represents the best technique and/or method to analyzed communication-alliance interactions since it offers both rigor and flexibility (Anguera et al., 2018), as proven by the broad range of observation tools created to analyze psychotherapy (e.g., Arias-Pujol and Anguera, 2017; Del Giacco et al., 2019) or other research areas (e.g., education, García-Fariña et al., 2018; sport, Tarragó et al., 2017). This scientific procedure, indeed, allows collecting qualitative data in observational records that are quantitized (Tashakkori and Teddlie, 1998) to obtain primary parameters (frequency, order, and duration) for carrying out quantitative analyses and identifying relationships between behaviors in systematized observational datasets (Anguera et al., 2017). In this study, we systematically observed the interactions between communication (as an integrated system of verbal and non-verbal dimensions) and the early TA construction in a group of depressed patients who show difficulties in developing and maintaining such a collaborative relationship because of their personality profile. For this purpose, we applied a peculiar and unconventional case of the observational method by developing a comprehensive procedure that integrates an *ad hoc* indirect observation system of verbal and non-verbal behaviors (the Communicative Modes Analysis System in Psychotherapy, CMASP; Del Giacco et al., 2018, 2019) and an observation instrument with deductive (or theoretical) categories for assessing the TA construction (the Collaborative Interactions Scale-Revised, CIS-R; Colli et al., 2014). Studies on such integration are limited and outdated (e.g., Bales and Cohen, 1979) and not focused on the interaction between communication and TA. In general, to our knowledge, no study has been conducted to observe systematically the micro-processes underlying the interaction of verbal (structures and intents) and non-verbal (voice and interruptions) communication with the TA construction in an Italian group of depressed patients by integrating a single observation system of communication with a tool based on deductive (or theoretical) categories for the alliance evaluation. We believe that this strategy may overcome the limits of previous research since it allows observing the complexity of mutual regulation processes between the therapist and the depressed patient from different perspectives at the same time.

Understanding the verbal and non-verbal communicative dynamics that promote the early TA construction between therapist and patients with depressive symptomatology can provide professionals with useful information to carry out interventions aimed, on the one hand, at containing the dysfunctional behavior of these patients and, on the other hand, at increasing the effectiveness of the therapy by laying the foundations for change. According to the previous theoretical background and the integration of two observational analysis techniques (lag sequential analysis and polar coordinate analysis) to obtain objective measures, this study aimed to analyze the action of specific communicative modes carried out by the therapist and depressed patients that foster the TA construction by each participant during the mutual regulation processes emerging in the initial stages of psychotherapy. Based on previous studies (Li et al., 2005; Dagnino et al., 2012; Tomicic et al., 2015b; Cafaro et al., 2016; Krause et al., 2016,^1^), we expect that the therapist's verbal (*asking* and *exploring*) and non-verbal (*elaborating* and *cooperatively interrupting*) modes and the depressed patients' verbal (*asserting* and *exploring*) and non-verbal (*expressing emotions* and *cooperatively interrupting*) modes positively affect the reciprocal construction of the early TA, determining stable patterns and significant associations with collaborative behaviors by each participant.

## Materials and Methods

We applied the observational methodology to carry out a systematic observation of the interactions between communication (verbal and non-verbal behaviors) and TA ruptures and repairs during the mutual regulation processes between therapist and depressed patients, based on an exploratory sequential mixed methods approach (Fetters et al., 2013). Starting from an initial exploratory analysis of the 20 psychotherapy sessions whereby the *ad hoc* indirect observation system CMASP was built (Del Giacco et al., 2019), in this study, we performed an in-depth study of the observational methodology by exploring sequential patterns and statistically significant relationships between communication and TA through the CMASP and CIS-R use. As we mentioned, the observational methodology (considered being mixed methods in itself) is intensive and involves working with a small number of participants, but it allows us to collect a large number of registers with high rigor (e.g., Arias-Pujol and Anguera, 2017; García-Fariña et al., 2018) by mixing qualitative (QUAL) and quantitative (QUANT) data (Plano Clark et al., 2015). Such a methodology establishes three ordered stages (QUAL-QUANT-QUAL) that can be complemented based on different options. Creswell and Plano Clark (2017) recommended this integration according to the *connecting* strategy in addition to the *merging* and *embedding* strategies. We believe that the first strategy (*connecting* by building a dataset on the other) is the most optimal one in this study, given the qualitative nature of our data that reveals their transformative capacity to facilitate the integration. Therefore, starting from the QUAL stage, we obtained a descriptive qualitative dataset through the non-participant and indirect observation of the initial sessions of psychotherapy that was transformed in a systematized register by using the CMASP and CIS-R. The integration between the *ad hoc* indirect observation system and the tool with deductive or (theoretical) categories provides information about verbal, vocal, and interruption behaviors (the CMASP) and TA variations in the form of ruptures and repairs (the CIS-R). Each recorded session, indeed, provides a matrix of codes where each row represents the observed unit that expresses the co-occurrence of behaviors related to the dimensions of the two instruments. According to a quantification record process, the observational methodology provides the primary parameters of frequency, order, and duration organized based on a progressive order of inclusion (Bakeman, 1978; Anguera et al., 2017): from frequency (which supplies the least information) to duration (which adds time units besides the other two). Specifically, “the order parameter is crucial for detecting hidden structures through the quantitative analysis of relationships between different codes in systematized observational datasets” (Anguera et al., 2017, p. 6). This parameter (which also comprises frequency) is essential in the quantitizing process of our study because it is suitable for the defined purposes and the nature of data. Therefore, in the second stage (QUANT stage), after having tested and passed the data quality control, it is possible to perform analyses through different quantitative techniques for categorical data (e.g., lag sequential analysis, polar coordinate analysis, and detection of T-Patterns) obtaining quantitative results that can be qualitatively interpreted in the third and last stage (QUAL stage) based on the research problem. All this leads to a perfect integration (Anguera et al., 2017).

### Design

The observational methodology provides eight observational designs deriving from the intersection of three dichotomous criteria (Blanco-Villaseñor et al., 2003; Portell et al., 2015): the unit of study, distinguished in *idiographic* (a single participant or a natural group of participants with a stable bond such as the family) and *nomothetic* (a group of participants) studies; the continuity of recording, divided into single-session (*point*) and multiple-session (*follow-up*) studies; and the level of response (or dimensionality), differentiated between *unidimensional* (a single level) and *multidimensional* (multiple levels) designs. Each one is characterized by an increasing level of complexity that leads the study in terms of data collection, organization, and analysis (Anguera et al., 2018). We employed a Nomothetic/Follow-up/Multidimensional (N/F/M; Blanco-Villaseñor et al., 2003) design because it showed the highest level of complexity and information that fitted the complexity of this research. It was *nomothetic* because we studied different participants (therapist-patient interaction in seven psychotherapies), *follow-up* because we collected data over seven clinical cases of three successive sessions each (inter-sessional follow-up) and recorded each whole session without interruption (intra-sessional follow-up), and *multidimensional* because we observed communication (verbal, vocal, and interruption behaviors) and TA (ruptures and repairs) as an integrated system of different dimensions.

### Participants and Materials

We selected the individual psychotherapies with 7 Italian university students (3 men and 4 women; age M = 26 years, SD = 3.91) self-referred to the Dynamic Psychotherapy Service (DPS) of the University of Padua (Italy) for problems of insecurity and difficulties in relationships and adaptation to the environment, low self-esteem, and deflected mood. They were treated by the same female therapist with 15 years of experience in brief focal psychotherapy, a form of once-a-week psychodynamic therapy lasting 15 sessions in which the therapist and patient develop the central focus of the treatment on a circumscribed problem area of discomfort for the latter during the initial assessment process (Rawson, 2018). Patients showed depressed symptomatology without psychiatric diagnosis detected through a previous screening to the assessment with the Beck Depression Inventory-II (BDI-II, Italian version; Ghisi et al., 2006) and the Symptom Checklist 90-Revised (SCL-90-R, Italian version; Sarno et al., 2011). The inclusion criteria for the patients' recruitment were (a) agreement to participate (signing the informed content to the research and tape recording), (b) initial assessment stage completed, (c) presence of depressive symptoms with scores ≥ 85^th^ percentile in all scales (Total Score, Somatic-Affective Area, and Cognitive Area) of the BDI-II and T scores ≥ 60 in the Global Severity Index and the Depression Scale of the SCL-90-R. The exclusion criteria were (a) psychiatric diagnosis, (b) ongoing pharmacological treatments for depression, (c) previous psychological treatments. Each case of psychotherapy comprised of 14 sessions of 50 min each. The sessions were entirely recorded by an MP3 recorder that was discreetly positioned in the therapy room at the same distance from the therapist and patient to minimize the reactivity bias. Based on the objectives of our research, we selected the audio recordings of the first three sessions of each clinical case (corresponding to the initial stage of psychotherapy) for a total of 21 sessions. Afterward, we eliminated one session audio recording because it was not complete (it stopped after 10 min), so the final sample was 20 sessions. Each audio recording was verbatim transcribed based on the norms defined by the CMASP manual (Del Giacco et al., 2018), which made it possible to produce a transcript that was also suitable for use with CIS-R. Therefore, we observed a total of 20 audio recordings and their corresponding transcripts equivalent to 6,232 speaking turns (3,121 therapist speaking turns + 3,111 patient speaking turns).

### Instruments

According to the systematic observation procedure (Anguera et al., 2018), recording instruments and the *ad hoc* observation instrument will be distinguished and described separately.

#### Recording Instruments

An MP3 audio recorder was used to record the psychotherapy sessions. We performed and used the verbatim transcription of each audio recording for indirect observation of verbal content. The Audacity® recording and editing software (v. 2.3.0; Audacity Team., 2018), a support instrument to listen, segment, trace, and code the audio tracks, was used to observe voice and interruption behaviors. We used Excel to report the codes of communication and TA.

#### Observational Instruments

##### The communicative modes analysis system in psychotherapy

The Communicative Modes Analysis System in Psychotherapy (CMASP; Del Giacco et al., 2018) is an *ad hoc* (Del Giacco et al., 2019) indirect observation system (Anguera et al., 2018) that determines the verbal, vocal, and interruption modes implemented by therapist and patient whereby they affect each other and co-construct meanings and psychological changes during communicative exchanges. It is a single classification system derived from the combination of two instruments of the observational method, the field format and category systems (Anguera et al., 2018), that is applied to audio recordings and verbatim transcripts and can be used at a global and dimension level (Table 1; for an in-depth description of the CMASP categories, see Supplementary Appendix I). The CMASP consists of four main dimensions based on the performative function of language (Searle, 2017): Verbal Mode-Structural Form (VeM-SF; six categories) and Verbal Mode-Communicative Intent (VeM-CI; eight categories) that evaluate the formal structure and communicative intent of verbal content, respectively; Vocal Mode (VoM; eight categories) that analyzes the communicative intent of the speaker's voice (regardless of verbal content) based on specific combinations of acoustic parameters impacting on the listener; Interruption Mode (IM; 11 categories) that identifies the interrupter's intent to support or hinder the communicative flow of the current speaker. This classification system comprises 33 categories derived from the observational method application and previous studies (Hill, 1978; Goldberg, 1990; Stiles, 1992; Murata, 1994; Li, 2001; Valdés et al., 2005, 2010; Krause et al., 2009; Tomicic et al., 2015a). Each dimension consists of a set of exhaustive and mutually exclusive (E/ME; Anguera et al., 2018) categories. The coder divides the audio recording and its verbatim transcript into speaking turns, each of which represents the unit of analysis. The verbatim transcript is the support to identify the structural form and communicative intent of verbal communication, while the audio recording to detect vocal and interruption modes through careful listening. The coder attributes to each speaking turn one and only one predominant communicative mode of each dimension.

**Table 1:** Summary scheme of the Communicative Modes Analysis System in Psychotherapy (CMASP) (retrieved from Del Giacco et al., 2019).

**Verbal Mode-Structural Form (VeM-SF)**	**Verbal Mode-Communicative Intent (VeM-CI)**	**Vocal Mode (VoM)**	**Interruption Mode (IM)**
Courtesies (SF1) Assertion (SF2) Question (SF3) Agreement (SF4) Denial (SF5) Direction (SF6)	Acknowledging (CI1) Informing (CI2) Exploring (CI3) Deepening (CI4) Focusing (CI5) Temporizing (CI6) Attuning (CI7) Resignifying (CI8)	Reporting (VM1) Connected (VM2) Declarative (VM3) Introspective (VM4) Emotional-Positive (VM5) Emotional-Negative (VM6) Pure Positive Emotion (VM7) Pure Negative Emotion (VM8)	Cooperative-Concurrence (IM1) Cooperative-Assistance (IM2) Cooperative-Clarification (IM3) Cooperative-Exclamation (IM4) Intrusive-Disagreement (IM5) Intrusive-Floor taking (IM6) Intrusive-Competition (IM7) Intrusive-Topic change (IM8) Intrusive-Tangentialization (IM9) Neutral interruption (IM10) Failed Interruption (IM11)

##### The collaborative interactions scale-revised

The Collaborative Interactions Scale-Revised (CIS-R; Colli et al., 2014) is the revised version of the CIS (Colli and Lingiardi, 2009), an observational tool with deductive or (theoretical) categories to assess ruptures and repairs of the TA through a micro-analytic evaluation of the therapeutic process (Table 2; for an in-depth description of the CIS-R categories, see Supplementary Appendix II). In this study, we used the CIS-R for a categorical coding by detecting the therapist's and depressed patients' ruptures and repairs at a speaking turn level. This transcript-based method, derived from Safran and Muran's (2003) theorization of TA, comprises two main scales for a total of 31 mutually exclusive and deductive categories: the Collaborative Interactions Scale-Therapist (CIS-T), to evaluate the therapist's positive and negative contributions to the therapeutic relationship, and the Collaborative Interactions Scale-Patient (CIS-P), to evaluate the patient's rupture and collaborative processes. The CIS-T is composed of the Form of the Therapist Intervention (TI) and the object of the therapist intervention. This last one is further divided into three subscales: Direct Collaborative Interventions (DCIs; four categories) and Indirect Collaborative Interventions (ICIs; three categories), the therapist's collaborative contributions directly or not directly related to the relationship with the patient or certain aspects of the therapy; and Rupture Interventions (RIs; five categories), the therapist's actions that negatively impact on the psychotherapy process. The CIS-P is composed of four subscales: Direct Collaborative Processes (DCPs; three categories) and Indirect Collaborative Processes (ICPs; three categories), the collaborative contributions to the TA construction directly or not directly related to the therapy and the therapeutic relationship; Direct Ruptures Markers (DRMs; four categories) and Indirect Rupture Markers (IRMs; five categories), the patient's ruptures of the TA directly or not directly related to the therapy. First, to evaluate the TA within a psychotherapy session, the transcript is divided into speaking turns, each of which represents the unit of analysis. Afterward, the speaking turns are grouped into narrative units, each one comprising a therapist-patient exchange. Finally, these are grouped into 10 homogeneous segments composing the psychotherapy session transcript.

**Table 2:** Summary scheme of the Collaborative Interactions Scale-Revised (CIS-R) (adapted from Colli et al., 2014).

**CIS-Therapist**	**CIS-Patient**
Form of Therapist Interventions (TI) Supportive (TI1) Explicative (TI2) Explorative (TI3) Expressive (TI4) Direct Collaborative Interventions (DCI) Task/Goal (DCI1) Affects (DCI2) Meaning (DCI3) Meta Communication (DCI4) Indirect Therapist Interventions (ICI) Facts (ICI1) Affects (ICI2) Meaning (ICI3) Rupture Interventions (RI) Linguistic Avoidance (RI1) Affective Avoidance (RI2) Hostility (RI3) Perseveration (RI4) Lack of Clarity (RI5)	Direct Collaborative Processes (DCP) Negotiation Tasks/Goals (DCP1) Affects (DCP2) Meaning (DCP3) Indirect Collaborative Processes (ICP) Facts (ICP1) Affects (ICP2) Meaning (ICP3) Direct Rupture Markers (DRM) Task/Goal (DRM1) Relationship (DRM2) Discouragement (DRM3) Parameters (DRM4) Indirect Rupture Markers (IRM) Linguistic Avoidance (IRM1) Affective Avoidance (IRM2) Self-esteem Regulation Strategies (IRM3) Indirect Allusions (IRM4) Acquiescence (IRM5)

*The authors granted permission to use the CIS-R scheme*.

As a first step, the coder performs a categorical coding by detecting ruptures or repairs that the therapist and patient implemented at a speaking turn level and attributing one and only one predominant category of the CIS-T or CIS-P, respectively. Afterward, it is possible to evaluate the TA trend within a psychotherapy session by applying a 4-point Likert scale to each coded category based on its frequency in all speaking turns of a segment. Moreover, it is possible to determine the intensity levels of ruptures and repairs for the therapist and patient, respectively, using a 3-point Likert scale at the global level. Finally, it is possible to obtain a TA global score for each psychotherapy session as a final result of the interactive processes between the ruptures and repairs of the therapist and patient.

#### Data Analysis Software

We used SPSS v. 23.0 statistics to perform the inter-rater reliability for the CIS-R and descriptive statistics. Moreover, the Generalized Sequential Querier computer program (GSEQ, v. 5.1.23; Bakeman and Quera, 2011) was used to carry out the intra-observer reliability for the CMASP and lag sequential analysis. Finally, we used the Tool for the Observation of Social Interaction in Natural Environments (HOISAN, v. 1.6.3.3.4; Hernández-Mendo et al., 2012) to perform the inter-observer reliability for the CMASP and the polar coordinate analysis.

### Procedure

As we mentioned previously, the 20 psychotherapy sessions audio recordings were first verbatim transcribed according to the norms defined by the CMASP manual (Del Giacco et al., 2018). Then, we segmented each audio recording and its transcript to divide them into meaningful units (Anguera et al., 2018) based on the study purposes. To do this, we applied Krippendorff's unitizing procedure that consists in performing “systematic distinctions within a continuum of otherwise undifferentiated text—documents, images, voices, videos, websites, and other observables– that are of interest to an analysis, omitting irrelevant matter but keeping together what cannot be divided without loss of meaning” (Krippendorff, 2018, p. 88). As a result of such a procedure, we defined the division of audio recordings and their transcripts into speaking turns, and each one represented our unit of analysis. A turn comprised any speech of a speaker that ended when the other participant took the floor, marked in the audio trace through Audacity® software (v. 2.3.0; Audacity Team., 2018). The CIS-R unitizing procedure produced the same segmentation as the CMASP; for this reason, we could use the speaking turn as the unit of analysis for both instruments and the transcript as single support to report their codes.

The 20 sessions (corresponding to the first three sessions, the initial stage, of each psychotherapy) were analyzed to data collection and analysis. Firstly, we administered the CMASP to each psychotherapy session: VeM-SFs and VeM-CIs were coded by analyzing each speaking turn in the transcript, while VoMs and IMs by carefully listening to speaking turn in the audio recording through the Audacity software (v. 2.3.0; Audacity Team., 2018). Following the coding manual (Del Giacco et al., 2018), we applied one dimension of the CMASP at a time to each speaking turn of the therapist and patients and attributed one and only one predominant communicative mode of the dimension considered. A systematized register of verbal (structures and intents), vocal, and interruption modes resulted in the form of a matrix of codes where each speaking turn expressed multiple event codes (Bakeman, 1978). Then, the CIS-R was administered to verbatim transcripts based on its coding procedures (Colli et al., 2014). Each speaking turn of the therapist and patients were analyzed by CIS-T and CIS-P, respectively, assigning one and only one predominant code for the ruptures or repairs used. A systematized register of ruptures and repairs resulted in the form of a catalog where each speaking turn expressed event-based sequential data (Bakeman, 1978).

Before quantification of data resulting from indirect observation, Krippendorff (2018) recommends a rigorous data quality control for preventing possible biases from skewing results (Anguera et al., 2018). According to this, we performed the two main quantitative techniques for evaluating the reliability of data: intra-observer reliability, the agreement level of an observer in coding of the same psychotherapy session at two different times; and the inter-observer reliability, the agreement level of at least three observers in coding of the same psychotherapy session at the same time. Precisely, we tested the intra-and inter-observer reliability for the CMASP and the inter-rater reliability for the CIS-R. Following the procedure, we carried out the reliability check on 10% of all the sessions coded corresponding to two psychotherapy sessions in our study. Therefore, four trained judges independently coded such two sessions (equivalent to 503 speaking turns) drawn at random from the sample. The intra-observer reliability was calculated as the average Cohen's κ (Cohen, 1960) through GSEQ (v. 5.1.23; Bakeman and Quera, 2011). The inter-observer reliability was computed using Krippendorff's canonical agreement coefficient (Cc; Krippendorff, 1980) through HOISAN (v. 1.6.3.3.4; Hernández-Mendo et al., 2012). Finally, the inter-rater reliability of the tool with deductive (or theoretical) categories, equivalent to the inter-observer agreement of observational methodology, was calculated as the average of Cohen's κ through SPSS v. 23 statistics. The CMASP showed an average κ of 0.98 and an average Cc of 94%, confirming almost perfect intra-and inter-observer reliability for κ ≥ 0.81 (Cohen, 1960) and Cc ≥ 81% (Krippendorff, 1980), respectively. The CIS-R presented an average κ of 0.79, indicating good inter-rater reliability (0.61 ≤ κ < 0.81; Cohen, 1960).

After passing the data quality control, we performed a re-categorization process by grouping the data of some basic categories of CMASP into macro-categories with more global characteristics and appropriate to the extent of the constructs under investigation. Based on the reviewed studies on communication-TA interaction, indeed, the concepts of explorative intent (Dagnino et al., 2012), emotional voice (Tomicic et al., 2015b) and cooperative/intrusive interruptions^1^ analyzed the reality of therapeutic exchanges at a more global level. Such re-categorization was possible since, in observational methodology, the everyday life of behavioral flow can be observed at different levels of *granularity* (Schegloff, 2000) “as a function of the possibilities ranging from most molar to most molecular” (Anguera, 2020, p. 52), characterized by greater interconnectedness (the molar level) or greater objectivity (the molecular level; Anguera, 2017), respectively. For this reason, we grouped the communicative intents Exploring (CI3), Deepening (CI4), and Focusing (CI5) within the macro-category Global Exploration (CIGE). The vocal categories Emotional-Positive (VM5) and Emotional-Negative (VM6), related to the expression of positive and negative emotions during verbalizations, were grouped in the macro-category Emotional (VME). Finally, we included all categories of interruptions related to cooperative and intrusive behaviors within the macro-categories Cooperative (IMC) and Intrusive (IMI), respectively.

**Figure 1:**
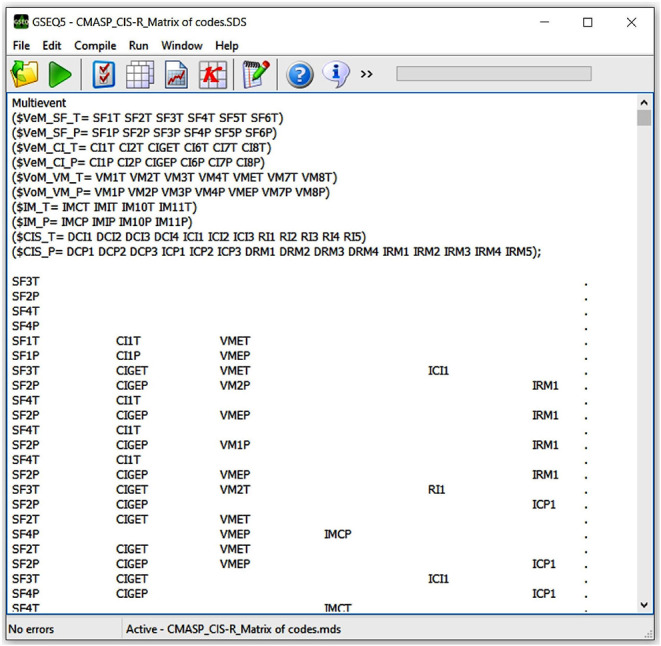
Screenshot of CMASP and CIS-R merged data in the form of a code matrix in GSEQ (v. 5.1.23; Bakeman and Quera, 2011). Each row corresponds to the multiple and concurrent event codes of a speaking turn. T and P distinguish the therapist's and patients' codes in their respective speaking turns.

Based on mixed methods approach, data resulting from CMASP and CIS-R application could then be merged in a comprehensive dataset (Fetters et al., 2013) since (a) their coding procedures fitted each other, (b) a predominant code could be attributed at a speaking turn level in both instruments, (c) the resulting data were categorical for both CMASP and CIS-R. Therefore, we obtained a systematized register of communicative modes and alliance ruptures and repairs in the form of a matrix of codes where each speaking turn of the therapist and depressed patients expressed multiple and co-occurrent event codes (Bakeman, 1978) of CMASP and CIS-R together (Figure 1).

### Statistical Analyses

We used three statistical analysis techniques to answer the study aim: descriptive statistics, lag sequential analysis, and polar coordinate analysis.

#### Descriptive Statistics

We performed a macro-analytical analysis through SPSS Statistics (v. 23.0) to describe quantitatively the communicative modes and the alliance ruptures and repairs used by the therapist and depressed patients during communicative exchanges.

#### Lag Sequential Analysis

We performed lag sequential analysis (Bakeman and Quera, 2011) to identify the stable behavioral patterns connected to the TA construction deriving from the action of specific communicative modes in the initial stages of psychotherapy. This statistical technique is used in observational methodology to analyze the sequences of behaviors detected through direct and/or indirect observation, being effective in different research areas (e.g., psychotherapy, Venturella et al., 2019; education, Santoyo et al., 2017; sport, Tarragó et al., 2017). The first step consists in establishing the *criterion behaviors* (i.e., the trigger behaviors of any possible pattern detected) and applying time lags defined for the study. Afterward, the observed probabilities of co-occurring *conditional behaviors* (i.e., associated behaviors) are calculated for each lag by using the binomial test; this test produces adjusted residuals (Z; Allison and Liker, 1982) that express the strength of association between significantly associated categories (i.e., between the criterion behaviors and the associated conditional behaviors). The significance level was fixed at *p* < 0.05. Adjusted residuals can be prospective or retrospective depending on whether the lags are analyzed in a forward (lag+1, lag+2, etc.) or backward (lag-1, lag-2, etc.) direction from the criterion behavior. They are statistically significant for values > 1.96 (excitatory association) and < −1.96 (inhibitory association) between criterion and conditional behaviors. To evaluate the strength of patterns, Bakeman and Gottman (1987) defined interpretative rules which conventionally establish that (a) a pattern ends when two or more consecutive lags present non-significant behaviors, (b) a pattern weakens when two successive lags exhibit multiple behaviors (the first one is the last interpretable, called Max Lag).

Based on the study aim and the reviewed literature, we selected the following communicative modes as criterion behaviors: Question (SF3), Global Exploration (CIGE), Connected (VM2), and Cooperative (IMC) for the therapist; Assertion (SF2), Global Exploration (CIGE), Emotional (VME), and Cooperative (IMC) for depressed patients. The alliance ruptures and repairs were assumed as conditional behaviors. We considered only the CMASP and CIS-R categories with a frequency > 5 since behavioral occurrences less than this value are not significant in observational methodology practice (Sackett, 1980). Ten retrospective lags (from lag −10 to lag-1) and 10 prospective lags (from the lag+1 to lag +10) were analyzed to investigate the associations between communication and the TA construction. This choice, while not involving the exploration of all possible lags, allows us to adequately catch the complexity of the research object, making progress compared to the usual practice of analyzing only five lags (Sackett, 1980). The GSEQ program (v. 5.1.23; Bakeman and Quera, 2011) was used on multiple and concurrent event data.

#### Polar Coordinate Analysis

**Figure 2:**
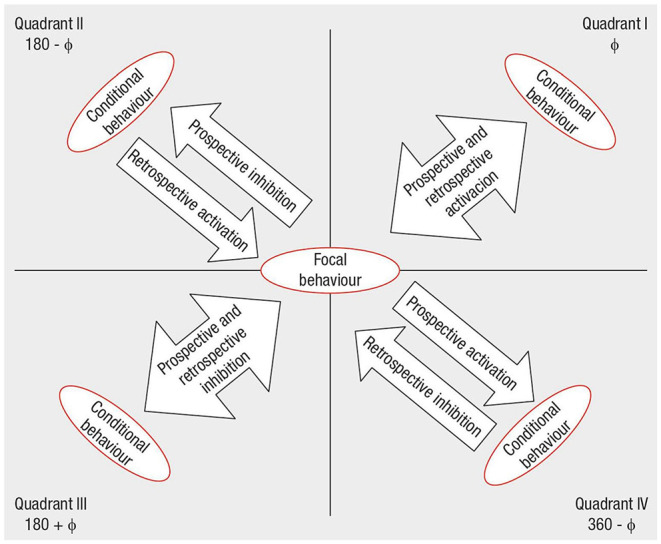
Polar coordinates' vector map that depicts the relationship between the focal and conditional behaviors, based on the quadrant where the vector is located (retrieved from Aragón et al., 2016, p. 5). The authors granted permission to use the image.

Polar coordinate analysis (Sackett, 1980; Anguera, 1997) identified the statistically significant relationships between one *focal behavior* (i.e., the behavior of interest) and *conditional behaviors* (i.e., associated behaviors). Such a quantitative analytical technique, widely used in different research areas (e.g., psychotherapy, Arias-Pujol and Anguera, 2017; education, Camerino et al., 2019; sport, Tarragó et al., 2017; interventions at the workplace, Portell et al., 2019), complements lag sequential analysis by reducing the volume of conditional probability data obtained by the latter through the Z_sum_ algorithm (Z_sum_ = $\frac{\;}{\sqrt{n}}$, where *Z* is the standard value of each adjusted residual deriving from the sequential analysis and *n* is the number of lags; Cochran, 1954). This statistic reflects the association between the focal behavior and each conditional behavior, and it is calculated for both prospective lags (Z_sum_ P, lags +1 to +5 or more) and retrospective lags (Z_sum_ R, lags −1 to −5 or less) (Sackett, 1980, 1987), obtaining a prospective and retrospective value for each conditional behavior. Anguera (1997) modified the original technique by introducing the concept of *genuine retrospectivity* to optimize the procedure. A vectorial depiction of the interrelationships between the focal behavior and each conditional behavior supports the analysis. Z_sum_ P and Z_sum_ R values are reported along the X and Y axes, respectively, defining the four quadrants of the vectors map where the focal behavior is the zero point (Figure 2). These values and the interaction between the positive or negative signs of Z_sum_ R and Z_sum_ P define the quadrant where each vector is located and its respective length (or radius) and angle (Sackett, 1980). The radius (Radius = $\sqrt{{\left(Z_{sum}P\right)}^{2}+{\left(Z_{sum}R\right)}^{2}}$) expresses the strength of the relationship and is statistically significant for values > 1.96 with *p* < 0.05. The angle (ϕ = $\frac{arcsine\;Z_{sum}\;R}{radius}\;$) shows the nature of the relationship and is adjusted as follows, depending on the quadrant where the vector is located: quadrant I (0° < ϕ <90°) = ϕ; quadrant II (90° < ϕ <180°) = 180° – ϕ; quadrant III (180° < ϕ <270°) = 180° + ϕ; quadrant IV (270° < ϕ <360°) = 360° – ϕ.

Each quadrant indicates the (inhibitory vs. excitatory) association between the focal and conditional behaviors: Quadrant I (+ +) expresses a mutually excitatory relationship between the focal and conditional behaviors (i.e., they activate each other); in Quadrant II (– +), the focal behavior inhibits and, at the same time, is activated by the conditional behavior; Quadrant III (– –) shows a mutually inhibitory relationship between the focal and conditional behaviors (i.e., they inhibit each other); and in Quadrant IV (+ –), the focal behavior activates and, at the same time, is inhibited by the conditional behavior.

We chose the communicative modes related to the study aim as focal behaviors [Question (SF3), Global Exploration (CIGE), Connected (VM2), and Cooperative (IMC) for the therapist; Assertion (SF2), Global Exploration (CIGE), Emotional (VME), and Cooperative (IMC) for depressed patients] and alliance ruptures and repairs as conditional behaviors. The polar coordinate analysis and vectorial maps were performed through the HOISAN program (v. 1.6.3.3.4; Hernández-Mendo et al., 2012) considering 10 lags (from lag −10 to lag −1) for Z_sum_ R and 10 lags (from lag+1 to lag +10) for Z_sum_ P.

## Results

Firstly, we introduce the general results of the descriptive statistics obtained by applying the CMASP and CIS-R. Then, we focus on the lag sequential analysis and polar coordinate analysis of the specific communicative modes implemented by the therapist and depressed patients that affect the reciprocal construction of a positive TA by each participant during the mutual regulation processes in the initial stages of psychotherapy.

### Main Communicative Modes Used by the Therapist and Depressed Patients

As shown in Table 3, from the comparison between the communicative modes used by the therapist and depressed patients during the initial stage of psychotherapy, the predominant structural forms characterizing their speech are Assertion (SF2), especially depressed patients, and Agreement (SF4) and Question (SF3), especially the therapist. The participants' verbal content mainly expresses communicative intents of Acknowledging (CI1), by taking the other's point of view about his/her experience (especially the therapist), and Global Exploration (CIGE) of his/her own or other's inner world (especially depressed patients). The vocal modes modulating the verbal content are mainly Connected (VM2), whereby participants perform elaborative processes in connection with themselves and oriented to the other (especially depressed patients), and Emotional (VME), whereby participants modulate speech through their emotional states (especially depressed patients). Finally, during communicative exchanges, participants mainly implement interruption modes of the type Cooperative (IMC) (especially the therapist).

**Table 3:** CMASP categories distribution in the therapist and depressed patients (*N* = 6,232 speaking turns).

	**Therapist** **(*n* = 3,121 speaking turns)**		**Patients group** **(*n* = 3,111 speaking turns)**	
**CMASP**	**f**	**%**	**f**	**%**
Verbal Mode-Structural Form (VeM-SF)	2,750	88.11	2,997	96.34
Courtesies (SF1)	23	0.84	29	0.97
Assertion (SF2)	832	30.25	2,467	82.32
Question (SF3)	687	24.98	65	2.17
Agreement (SF4)	1,149	41.78	366	12.21
Denial (SF5)	11	0.40	69	2.30
Direction (SF6)	48	1.75	1	0.03
Not coded	371	11.89	114	3.66
Verbal Mode-Communicative Intent (VeM-CI)	2,503	80.20	2,668	85.76
Acknowledging (CI1)	1,108	44.27	167	6.26
Informing (CI2)	140	5.59	56	2.10
Global Exploration (CIGE)	832	33.24	2,202	82.53
Temporizing (CI6)	3	0.12	23	0.86
Attuning (CI7)	180	7.19	47	1.76
Resignifying (CI8)	240	9.59	173	6.48
Not coded	618	19.80	443	14.24
Vocal Mode (VoM)	1,419	45.47	2,413	77.56
Reporting (VM1)	2	0.14	8	0.33
Connected (VM2)	670	47.22	851	35.27
Declarative (VM3)	92	6.48	87	3.61
Introspective (VM4)	9	0.63	177	7.34
Emotional (VME)	339	23.89	1,214	50.31
Pure Positive Emotion (VM7)	287	20.23	46	1.91
Pure Negative Emotion (VM8)	20	1.41	30	1.24
Not coded	1,702	54.53	698	22.44
Interruption Mode (IM)	550	17.62	585	19.09
Cooperative (IMC)	238	43.27	209	35.19
Intrusive (IMI)	171	31.09	180	30.30
Neutral Interruption (IM10)	96	17.45	190	31.99
Failed Interruption (IM11)	45	8.18	15	2.53
Not coded	2,571	82.38	2,526	81.20

### Alliance Ruptures and Repairs Used by the Therapist and Depressed Patients

In Table 4, it is possible to notice that, during the initial phase of the psychotherapy, the therapist above all contributes to the TA through Indirect Collaborative Interventions (ICI) focused on Facts (ICI1), Affects (ICI2), and Meaning (ICI3) related to the depressed patients' experiences and through Direct Collaborative Interventions (DCI) related to the Task/Goals of the therapy (DCI1). Moreover, the therapist tends to break the TA through Rupture Interventions (RI), mainly characterized by suddenly changing the topic in the form of Linguistic Avoidance (RI1) and by Hostility (RI3). On the other hand, depressed patients contribute to TA construction through Indirect Collaborative Processes (ICP) related to Facts (ICP1) and Affects (ICP2). Moreover, they implement Indirect Rupture Markers (IRM) characterized by Linguistic Avoidance (IRM1) and Affective Avoidance (IRM2).

**Table 4:** CIS-T and CIS-P items distribution (*N* = 6,232 speaking turns).

	**Therapist (*****n*** **= 3,121 speaking turns)**			**Patients group (*****n*** **= 3,111 speaking turns)**	
**CIS-R**	***f***	**%**		***f***	**%**
CIS-Therapist (CIS-T)	1,215	38.93	CIS-Patient (CIS-P)	2,529	81.29
Direct Therapist Intervention (DCI)	165	13.58	Direct Collaborative Processes (DCP)	98	3.88
Task/Goal (DCI1)	137	11.28	Negotiation Tasks/Goals (DCP1)	48	1.90
Affects (DCI2)	19	1.56	Affects (DCP2)	48	1.90
Meaning (DCI3)	9	0.74	Meaning (DCP3)	2	0.08
Meta communication (DCI4)	0	0.00	Indirect Collaborative Processes (ICP)	1,106	43.73
Indirect Therapist Intervention (ICI)	787	64.77	Facts (ICP1)	786	31.08
Facts (ICI1)	455	37.45	Affects (ICP2)	227	8.98
Affects (ICI2)	177	14.57	Meaning (ICP3)	93	3.68
Meaning (ICI3)	155	12.76	Direct Rupture Marker (DRM)	40	1.58
Rupture Interventions (RI)	263	21.65	Task/Goal (DRM1)	2	0.08
Linguistic Avoidance (RI1)	140	11.52	Relationship (DRM2)	35	1.38
Affective Avoidance (RI2)	0	0.00	Discouragement (DRM3)	0	0.00
Hostility (RI3)	122	10.04	Parameters (DRM4)	3	0.12
Perseveration (RI4)	1	0.08	Indirect Rupture Marker (IRM)	1,285	50.81
Lack of Clarity (RI5)	0	0.00	Linguistic Avoidance (IRM1)	798	31.55
Not coded	1,906	61.07	Affective Avoidance (IRM2)	337	13.33
			Self-esteem Regulation Strategies (IRM3)	43	1.70
			Indirect Allusions (IRM4)	26	1.03
			Acquiescence (IRM5)	81	3.20
			Not coded	582	18.71

### Behavioral Patterns of Depressed Patients and Therapist in the Therapeutic Alliance Construction

Tables 5–**8** show the sequential patterns of behaviors related to the TA construction in the therapist and depressed patients considering the communicative modes detected from the reviewed literature as criterion behaviors. We have structured the results into sections organized by the different four verbal and non-verbal dimensions that have been analyzed for the therapist and depressed patients. We will discuss only the behavioral patterns with Z values > 1.96 (*p* < 0.05), representing the excitatory relationships between criterion and conditional behaviors.

**Table 5:** Depressed patients' and therapist's behavioral patterns in the alliance construction due to the action of the reciprocal structural forms.

**Lag** **−10**	**Lag** **−9**	**Lag** **−8**	**Lag** **−7**	**Lag** **−6**	**Lag** **−5**	**Lag** **−4**	**Lag** **−3**	**Lag** **−2**	**Lag** **−1**	**CB**	**Lag** **+1**	**Lag** **+2**	**Lag** **+3**	**Lag** **+4**	**Lag** **+5**	**Lag** **+6**	**Lag** **+7**	**Lag** **+8**	**Lag** **+9**	**Lag** **+10**
**ICP1** **(3.69)**	ICP1 (4.77)	ICP1 (5.80)	ICP1 (3.10)	ICP1 (3.94)	ICP1 (5.28)	ICP1 (3.27)	ICP1 (6.98)	ICP1 (3.23)	ICP1 (6.70)	SF3T	ICP1 (9.60)	ICP1 (2.85)	ICP1 (7.21)	DCP1 (2.81)	ICP1 (6.33)	ICP2 (3.86)	ICP1 (5.89)	ICP2 (2.87)	ICP1 (5.19)	**ICP1** **(2.49)**
						ICP2 (2.79)								ICP1 (2.75)		DCP1 (2.52)			ICP2 (2.10)	
*IRM1* *(−3.52)*	*IRM1* *(−2.63)*	*IRM1* *(−2.59)*	*IRM1* *(−2.52)*	*IRM1* *(−2.33)*	*IRM1* *(−4.38)*	*IRM5* *(−2.31)*	*IRM1* *(−5.56)*	*ICP3* *(−2.39)*	***IRM1*** ***(–4.84)***		*IRM1* *(−5.05)*	*IRM1* *(−4.20)*	*IRM5* *(−4.37)*	*IRM1* *(−2.93)*	***IRM1*** ***(–4.18)***	*IRM1* *(−2.68)*	*IRM1* *(−2.96)*	*IRM1* *(−2.90)*	*IRM1 (−3.87)*	*DRM2* *(−2.24)*
	*IRM5* *(−2.12)*		*IRM5* *(−2.51)*	*IRM5* *(−1.98)*	*DCP2* *(−2.31)*	*IRM1* *(−2.20)*	*IRM5* *(−2.17)*	*DRM2* *(−2.16)*	***IRM5*** ***(–2.10)***		*IRM2* *(−3.14)*		*IRM1* *(−4.36)*		***IRM5*** ***(–3.22)***	*IRM5* *(−2.68)*	*IRM5* *(−2.91)*	*DRM2* *(−2.06)*	*IRM5* *(−3.20)*	
						*DRM2* *(−2.16)*					*IRM5* *(−2.74)*						*DRM2* *(−2.06)*		*ICP3* *(−2.13)*	
																			*DRM2* *(−2.00)*	
**ICI1** **(3.27)**	ICI1 (4.20)	ICI1 (3.84)	ICI1 (4.89)	ICI1 (3.70)	ICI1 (5.52)	ICI1 (3.18)	ICI1 (7.49)	ICI1 (2.52)	ICI1 (8.57)	SF2P	ICI1 (9.43)	ICI1 (3.83)	ICI1 (7.81)	ICI1 (4.35)	ICI1 (5.41)	ICI2 (2.34)	ICI1 (4.41)	ICI1 (2.93)	ICI1 (5.50)	**ICI1** **(2.33)**
					ICI2 (2.92)		ICI2 (4.58)		ICI2 (4.64)		ICI2 (3.42)		ICI2 (3.96)		ICI2 (2.61)					
*DCI1* *(−3.31)*	*DCI1* *(−5.10)*	*DCI1* *(−4.16)*	*DCI1* *(−7.14)*	*DCI1* *(−4.12)*	*DCI1* *(−6.47)*	*DCI1* *(−4.71)*	*DCI1* *(−7.63)*	*ICI3* *(−2.73)*	***DCI1*** ***(–8.88)***		*ICI3 (−7.64)*	*DCI1* *(−3.89)*	***DCI1*** ***(–7.40)***	*DCI1* *(−3.60)*	*DCI1* *(−6.33)*	*DCI1* *(−3.16)*	*DCI1* *(−3.82)*	*DCI1* *(4.02)*	*DCI1* *(−3.06)*	*DCI1* *(−3.63)*
*ICI3* *(−3.25)*	*RI1* *(−2.45)*	*ICI3* *(−3.37)*	*RI1* *(−2.46)*		*ICI3* *(−3.35)*		*ICI3* *(−4.83)*	*DCI3* *(−2.27)*	***ICI3*** ***(–7.78)***		*DCI1* *(−7.32)*		***ICI3 (–4.16)***	*ICI3* *(−2.74)*	*ICI3* *(3.17)*	*ICI3* *(−3.17)*	*ICI3* *(−2.30)*		*ICI3* *(−2.83)*	
*ICI3* *(−2.02)*					*RI1* *(−2.56)*		*RI1* *(−2.93)*						***RI3*** ***(–2.75)***	*DCI2* *(−2.23)*						
													***DCI2*** ***(–1.98)***							

***Structural Form (Therapist)-CIS (Patient) Interaction**: Criterion Behavior (CB): structural form Question (SF3T); Conditional Behaviors: Direct Collaborative Processes on Negotiation Tasks/Goals (DCP1) and Affects (DCP2); Indirect Collaborative Processes on Facts (ICP1), Affects (ICP2), and Meaning (ICP3); Direct Rupture Markers on Relationship (DRM2); Indirect Rupture Markers as Linguistic Avoidance (IRM1), Affective Avoidance (IRM2), and Acquiescence (IRM5). **Structural Form (Patient)-CIS (Therapist) Interaction**: Criterion Behavior (CB): structural form Assertion (SF2P); Conditional behaviors: Direct Collaborative Interventions on Task/Goal (DCI1), Affects (DCI2), and Meaning (DCI3); Indirect Therapist Interventions on Facts (ICI1), Affects (ICI2), and Meaning (ICI3); Rupture Interventions as Linguistic Avoidance (RI1) and Hostility (RI3). Z values > 1.96 indicate the excitatory relationships; Z values < -1.96 (in italics) indicate the inhibitory relationships; categories in bold indicate the Max lag and the end of the pattern; significance level at p <0.05*.

#### Verbal Mode-Structural Form

In Table 5, during the TA construction, the therapist's use of questions (SF3T) is followed and preceded with high probability by stable behavioral patterns of depressed patients expressed through collaborative processes related to the events experienced (ICP1). Moreover, such patients symmetrically activate collaborative processes on feelings and/or thoughts related to their experiences (ICP2), and only prospectively, collaborative processes focused on the therapy goals (DCP1).

Example:

*Patient*: This time, I decided not to stay home but to go out. (ICP1)
*Therapist*: How did you spend the day? (SF3T)
*Patient*: I went to the mountains with my girlfriend. (ICP1)

On the other hand, in the presence of assertions from depressed patients (SF2P), the therapist implements a stable and symmetrical pattern of collaborative interventions focused on patients' experiences (ICI1), supplemented by interventions on their feelings and/or thoughts (ICI2) in the lags immediately before and after the criterion behavior.

Example:

*Therapist*: Can you tell me something about your father? (ICI1)
*Patient*: My daddy grew up in Sicily, and when he speaks, he always gesticulates… (SF2P)
*Therapist*: For example,…. when does it happen? (ICI1)

#### Verbal Mode-Communicative Intent

In Table 6, the communicative intent Global Exploration (CIGET) -exploring, deepening, and focusing- of the therapist is followed and preceded with high probability by a stable pattern of depressed patients' collaborative processes related to the events experienced (ICP1); in prospective lags, such patients also activate collaborative processes on feelings and/or thoughts related to their experiences (ICP2).

**Table 6:** Depressed patients' and therapist's behavioral patterns in the alliance construction due to the action of the reciprocal communicative intents.

**Lag** **−10**	**Lag** **−9**	**Lag** **−8**	**Lag** **−7**	**Lag** **−6**	**Lag** **−5**	**Lag** **−4**	**Lag** **−3**	**Lag** **−2**	**Lag** **−1**	**CB**	**Lag** **+1**	**Lag** **+2**	**Lag** **+3**	**Lag** **+4**	**Lag** **+5**	**Lag** **+6**	**Lag** **+7**	**Lag** **+8**	**Lag** **+9**	**Lag** **+10**
**ICP1** **(3.73)**	ICP1 (3.66)	ICP1 (4.28)	ICP1 (4.72)	IRM3 (3.59)	ICP1 (5.96)	ICP1 (4.26)	ICP1 (7.31)	ICP1 (6.75)	ICP1 (8.53)	CIGET	ICP1 (11.37)	ICP1 (5.97)	ICP1 (7.24)	ICP1 (4.86)	ICP1 (5.87)	ICP1 (3.56)	ICP1 (4.90)	ICP1 (3.64)	ICP1 (5.96)	**ICP1** **(4.21)**
				ICP1 (3.50)		ICP2 (2.06)							ICP2 (2.23)			ICP2 (2.45)		ICP2 (2.53)		**ICP2** **(3.14)**
*IRM1* *(−2.89)*	*DCP2 (−2.19)*	*IRM1* *(−2.69)*	*IRM5* *(−2.71)*	*IRM1* *(−2.36)*	*IRM1* *(−3.77)*	*DCP1* *(−2.71)*	*IRM1* *(−5.30)*	*ICP3* *(−3.48)*	***IRM1*** ***(−4.57)***		***IRM1*** ***(−5.28)***	*IRM1* *(−5.28)*	*IRM1* *(−4.38)*	*IRM1* *(−3.73)*	*IRM1* *(−4.15)*	*IRM1* *(−3.74)*	*DRM2 (−2.62)*	*IRM1* *(−2.59)*	*IRM1* *(−3.18)*	*IRM1* *(−3.20)*
*DCP2 (−2.39)*		*IRM5* *(−2.24)*	*IRM1* *(−2.20)*	*ICP3* *(−2.03)*	*IRM5* *(−2.21)*	*IRM1* *(−2.64)*	*DCP1* *(−2.98)*	*DCP1* *(−2.75)*	***DCP1*** ***(−2.26)***		***IRM2*** ***(−3.21)***	*DCP1* *(−3.64)*	*DCP1* *(−3.05)*	*DCP1* *(−2.58)*	*DRM2 (−2.06)*		*IRM2* *(−2.07)*	*DRM2* *(−2.43)*	*DRM2 (−2.89)*	*IRM5* *(−2.69)*
*IRM5* *(−2.38)*		*DCP2* *(−2.14)*				*DCP2* *(−2.48)*	*IRM5* *(−2.43)*	*DRM2 (−2.22)*	***IRM5*** ***(−2.12)***		***ICP3*** ***(−2.54)***	*IRM5 (−2.63)*	*IRM5* *(−2.35)*	*IRM5* *(−2.20)*			*DCP1* *(−1.97)*	*IRM1* *(−2.40)*	*DCP1* *(−2.86)*	*DRM2* *(−2.18)*
						*DRM2* *(−2.37)*	*DCP2* *(−2.07)*	*IRM1* *(−2.15)*			***IRM5*** ***(−2.50)***								*IRM2* *(−1.97)*	
											***DCP1*** ***(−2.29)***									
**ICI1** **(6.04)**	ICI1 (5.77)	ICI1 (6.02)	ICI1 (6.67)	ICI1 (6.96)	ICI1 (9.10)	ICI1 (6.58)	ICI1 (10.82)	ICI1 (5.88)	ICI1 (12.30)	CIGEP	ICI1 (10.20)	ICI1 (6.25)	ICI1 (7.99)	ICI1 (4.94)	ICI1 (6.72)	ICI1 (3.58)	ICI1 (4.74)	ICI1 (4.25)	ICI1 (4.56)	**ICI1** **(4.47)**
		ICI2 (2.34)			ICI2 (2.02)				ICI2 (3.71)		ICI2 (2.54)		ICI2 (2.86)			IC2 (2.13)				
*DCI1* *(−7.30)*	*DCI1 (−6.63)*	*DCI1* *(−8.61)*	*DCI1* *(−7.67)*	*DCI1 (−6.98)*	*DCI1* *(−7.75)*	*DCI1 (−6.37)*	*DCI1* *(−8.14)*	*DCI1* *(−6.73)*	***DCI1*** ***(−9.70)***		***ICI3*** ***(−8.67)***	*DCI1* *(−5.46)*	*ICI3* *(−5.68)*	*ICI3* *(−4.30)*	*DCI3* *(−5.17)*	*ICI3* *(−4.98)*	*ICI3* *(−4.38)*	*ICI3* *(−4.70)*	*ICI3* *(−2.96)*	*ICI3* *(−5.81)*
*ICI3* *(−4.11)*	*ICI3* *(−2.97)*	*ICI3* *(−2.21)*	*DCI3* *(−3.32)*	*DCI3* *(−2.95)*	*ICI3* *(−5.03)*	*DCI3* *(−4.10)*	*ICI3* *(−6.37)*	*ICI3* *(−3.34)*	***ICI3*** ***(−9.57)***		***DCI1*** ***(−7.44)***	*ICI3* *(−3.64)*	*DCI1* *(−5.20)*	*DCI1* *(−3.84)*	*ICI3* *(−4.58)*	*DCI2* *(−2.32)*	*DCI3* *(−3.18)*	*DCI3* *(−2.48)*	*DCI3* *(−2.94)*	*DCI1* *(−2.93)*
*DCI3* *(−2.62)*	*DCI3* *(−2.73)*	*DCI3* *(−2.12)*	*ICI3* *(−2.71)*		*DCI3* *(−2.67)*	*DCI2* *(−2.15)*	*RI1* *(−2.91)*		***RI1*** ***(−4.29)***		***DCI3*** ***(−2.75)***	*DCI2* *(−3.52)*	*DCI3* *(−3.70)*	*DCI3* *(−3.52)*	*DCI1* *(−3.03)*	*DCI1 (−2.23)*			*DCI2* *(−2.47)*	
	*RI1* *(−1.99)*	*DCI2* *(−1.99)*	*RI1* *(−2.42)*		*RI1* *(−2.33)*		*DCI3* *(−2.63)*					*DCI3* *(−2.58)*	*RI3* *(−2.69)*		*DCI2* *(−2.84)*	*DCI3* *(−2.09)*			*DCI1* *(−2.05)*	
					*DCI2* *(−2.01)*		*DCI2* *(−2.33)*								*RI3* *(−2.11)*					

***Communicative Intent (Therapist)-CIS (Patient) Interaction**: Criterion Behavior (CB): communicative intent Global Exploration (CIGET); Conditional Behaviors: Direct Collaborative Processes on Negotiation Tasks/Goals (DCP1) and Affects (DCP2); Indirect Collaborative Processes on Facts (ICP1), Affects (ICP2), and Meaning (ICP3); Direct Rupture Markers on Relationship (DRM2); Indirect Rupture Markers as Linguistic Avoidance (IRM1), Affective Avoidance (IRM2), Self-esteem Regulation Strategies (IRM3), and Acquiescence (IRM5). **Communicative Intent (Patient)-CIS (Therapist) Interaction**: Criterion Behavior (CB): communicative intent Global Exploration (CIGEP); Conditional behaviors: Direct Collaborative Interventions on Task/Goal (DCI1), Affects (DCI2), and Meaning (DCI3); Indirect Therapist Interventions on Facts (ICI1), Affects (ICI2), and Meaning (ICI3); Rupture Interventions as Linguistic Avoidance (RI1) and Hostility (RI3). Z values > 1.96 indicate the excitatory relationships; Z values < -1.96 (in italics) indicate the inhibitory relationships; categories in bold indicate the Max lag and the end of the pattern; significance level at p <0.05*.

Example:

*Patient*: We're trying to sell the house because it's too expensive for one person. (ICP1)
*Therapist*: There's also, um, a difficult choice, that is, this choice to leave the house… (CIGET)
*Patient*: No, no, um, we're not…my sister and I aren't going to be there anymore. (ICP1)

Symmetrically, when depressed patients express the speech with the communicative intent Global Exploration (CIGEP), the therapist is likely to activate a stable pattern that precedes and follows such a criterion behavior, characterized by collaborative interventions on patients' experiences (ICI1) that are supplemented by interventions on their feelings and/or thoughts (ICI2).

Example:

*Therapist*: How's your relationship now? (ICI1)
*Patient*: Well, there's…um… respect between my boyfriend and me. (CIGEP)
*Therapist*: Do you still work together? (ICI1)

#### Vocal Mode

In Table 7, in the presence of the therapist's elaborative vocal mode (VM2T), depressed patients retrospectively activate (up to delay −3) collaborative processes on feelings and/or thoughts related to their experiences (ICP2), and prospectively (up to delay +3), collaborative processes related to the events experienced (ICP1), the therapy goals (DCP1), and their feelings toward the therapist and therapy (DCP2).

**Table 7:** Depressed patients' and therapist's behavioral patterns in the alliance construction due to the action of the reciprocal vocal modes.

**Lag** **−10**	**Lag** **−9**	**Lag** **−8**	**Lag** **−7**	**Lag** **−6**	**Lag** **−5**	**Lag** **−4**	**Lag** **−3**	**Lag** **−2**	**Lag** **−1**	**CB**	**Lag** **+1**	**Lag** **+2**	**Lag** **+3**	**Lag** **+4**	**Lag** **+5**	**Lag** **+6**	**Lag** **+7**	**Lag** **+8**	**Lag** **+9**	**Lag** **+10**
ICP3 (2.01)	ICP2 (2.62)					**ICP2** **(2.05)**	ICP2 (2.35)	ICP2 (2.52)	ICP2 (2.95)	VM2T	ICP1 (2.89)	ICP1 (2.53)	**DCP2** **(2.35)**			IRM3 (2.07)				DRM2 (2.09)
											DCP1 (2.57)									DCP1 (2.01)
*DCP1* *(−2.45)*				***IRM5*** ***(−2.80)***		*DCP1* *(−2.71)*	*DRM2* *(−2.13)*	*DRM2* *(−2.00)*	*IRM1* *(−2.97)*		*IRM1* *(−4.20)*	*IRM1* *(−2.26)*	***IRM1*** ***(−2.18)***							
						*IRM5* *(−2.13)*														
		ICI3 (2.07)					**ICI2** **(2.00)**	ICI2 (2.40)	DCI2 (2.95)	VMEP	ICI2 (2.80)	ICI2 (2.09)	**DCI2** **(2.15)**			ICI3 (2.60)	ICI3 (2.57)	ICI3 (3.17)	ICI3 (2.54)	ICI3 (2.06)
																	DCI2 (2.12)		DCI2 (2.51)	DCI2 (2.06)
	*DCI1* *(−2.27)*			*DCI3* *(−2.28)*			***DCI3*** ***(−2.42)***	*ICI1* *(−1.99)*						*RI1* *(−2.24)*				*ICI2* *(−2.20)*	*ICI1* *(−2.34)*	
				*ICI2* *(−1.99)*																

***Vocal Mode (Therapist)-CIS (Patient) Interaction**: Criterion Behavior (CB): vocal mode Connected (VM2T); Conditional Behaviors: Direct Collaborative Processes on Negotiation Tasks/Goals (DCP1) and Affects (DCP2); Indirect Collaborative Processes on Facts (ICP1), Affects (ICP2), and Meaning (ICP3); Direct Rupture Markers on Relationship (DRM2); Indirect Rupture Markers as Linguistic Avoidance (IRM1), Self-esteem Regulation Strategies (IRM3), and Acquiescence (IRM5). **Vocal Mode (Patient)-CIS (Therapist) Interaction**: Criterion Behavior (CB): vocal mode Emotional (VME); Conditional behaviors: Direct Collaborative Interventions on Task/Goal (DCI1), Affects (DCI2), and Meaning (DCI3); Indirect Therapist Interventions on Facts (ICI1), Affects (ICI2), and Meaning (ICI3); Rupture Interventions as Linguistic Avoidance (RI1). Z values > 1.96 indicate the excitatory relationships; Z values < −1.96 (in italics) indicate the inhibitory relationships; categories in bold indicate the Max lag and the end of the pattern; significance level at p <0.05*.

Example (from the audio track coding):

*Patient*: I feel happy when I listen to music! (ICP2)
*Therapist*: Last time, you were telling me that this is your biggest passion… (pause). (VM2T)
*Patient*: Yes! … I started late because I was 18 years old, but it was love at first sight. (ICP1)

On the other hand, in the presence of the depressed patients' emotional vocal mode (VMEP), the therapist symmetrically activates (up to lags −3 and +3) a pattern of collaborative interventions on feelings and/or thoughts of patients linked to their experiences (ICI2), integrated by collaborative interventions related to the patients' feelings toward the therapy and the therapist (DCI2).

Example (from the audio track coding):

*Therapist*: Wouldn't you have liked…to…to go to Japan too? (ICI2)
*Patient*: I think I'd be a different person with that kind of experience in Japan! (VMEP)
*Therapist*: Uhm! And what kind of person do you think you would be? (ICI2)

#### Interruption Mode

In Table 8, the therapist's use of cooperative interruption modes (IMCT) is followed and preceded with high probability by a stable pattern of depressed patients' collaborative processes related to the events experienced (ICP1). Moreover, such patients symmetrically activate collaborative processes on feelings and/or thoughts related to their experiences (ICP2), and only prospectively, collaborative processes related to the deep meaning of the events experienced (ICP3).

**Table 8:** Depressed patients' and therapist's behavioral patterns in the alliance construction due to the action of the reciprocal interruption modes.

**Lag** **−10**	**Lag** **−9**	**Lag** **−8**	**Lag** **−7**	**Lag** **−6**	**Lag** **−5**	**Lag** **−4**	**Lag** **−3**	**Lag** **−2**	**Lag** **−1**	**CB**	**Lag** **+1**	**Lag** **+2**	**Lag** **+3**	**Lag** **+4**	**Lag** **+5**	**Lag** **+6**	**Lag** **+7**	**Lag** **+8**	**Lag** **+9**	**Lag** **+10**
ICP2 (2.57)		ICP2 (2.24)			**ICP1** **(2.74)**	ICP1 (3.27)	ICP1 (2.51)	ICP1 (2.07)	ICP1 (3.60)	IMCT	ICP1 (3.60)	ICP1 (2.71)	ICP1 (2.96)	ICP1 (1.98)	ICP3 (2.02)	**ICP2** **(2.80)**				
					*IRM5* *(−2.11)*	*IRM2* *(−2.06)*			***DCP1*** ***(−3.07)***		*IRM5* *(−2.17)*		*DCP1* *(−2.05)*		*IRM1* *(−2.13)*	***IRM1*** ***(−2.08)***				
			**ICI2** **(3.27)**		DCI1 (2.36)	DCI1 (3.03)	DCI1 (2.62)	DCI1 (2.62)	DCI1 (3.70)	IMCP	DCI1 (3.27)	DCI1 (2.45)	DCI1 (2.90)	DCI1 (2.65)	DCI1 (2.04)		**DCI1** **(2.15)**			
			**DCI1** **(2.26)**						ICI3 (2.68)		ICI3 (3.24)						**DCI2** **(1.99)**			
		*RI1* *(−2.80)*	*ICI1* *(−2.41)*						***ICI1*** ***(−3.44)***		*RI3* *(−4.24)*		*ICI1* *(−2.21)*	***ICI2*** ***(−2.06)***						

***Interruption Mode (Therapist)-CIS (Patient) Interaction**: Criterion Behavior (CB): interruption mode Cooperative (IMCT); Conditional Behaviors: Direct Collaborative Processes on Negotiation Tasks/Goals (DCP1) and Affects (DCP2); Indirect Collaborative Processes on Facts (ICP1), Affects (ICP2), and Meaning (ICP3); Direct Rupture Markers on Relationship (DRM2); Indirect Rupture Markers as Linguistic Avoidance (IRM1), Self-esteem Regulation Strategies (IRM3), and Acquiescence (IRM5). **Interruption Mode (Patient)-CIS (Therapist) Interaction**: Criterion Behavior (CB): interruption mode Cooperative (IMCP); Conditional behaviors: Direct Collaborative Interventions on Task/Goal (DCI1), Affects (DCI2); Indirect Therapist Interventions on Facts (ICI1), Affects (ICI2), and Meaning (ICI3); Rupture Interventions as Linguistic Avoidance (RI1) and Hostility (RI3). Z values > 1.96 indicate the excitatory relationships; Z values < −1.96 (in italics) indicate the inhibitory relationships; categories in bold indicate the Max lag and the end of the pattern; significance level at p <0.05*.

Example (from the audio track coding):

*Patient:* I wasn't feeling well, so I made up an… an…ex// (interrupted) (ICP1)
*Therapist*: //an excuse? (IMCT)
*Patient*: Yes… but in the end, I told her the truth, and she was very understanding of me. (ICP1)

On the other hand, in the presence of a cooperative interruption mode by depressed patients (IMCP), the therapist activates with high probability a stable pattern of collaborative interventions focused on the therapy goals and tasks (DCI1). Such behaviors of the therapist are symmetrically integrated by interventions related to the meaning of patients' experiences (ICI3), retrospectively, by interventions on feelings and/or thoughts of patients about their experiences (ICI2), and prospectively, by interventions on patients' feelings toward the therapy and/or the therapist (DCI2).

Example (from the audio track coding):

*Therapist*: If you agree, I'd like to meet you for a few sessions to discuss your problems together and see how to proceed// (interrupted) (DCI1)
*Patient*: //What do you mean “how to proceed”? (IMCP)
*Therapist*: What to advise you on, how to deal with your difficulties… (DCI1)

### Relationships Between the Communicative Modes and the Construction of the Therapeutic Alliance

**Figure 3:**
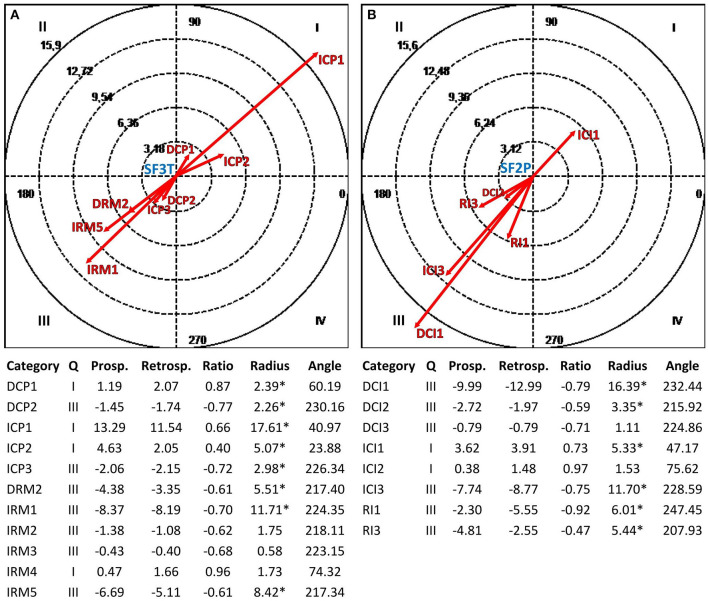
Vectorial maps of the statistically significant relationships for the therapist **(A)**, considering the structural form Question (SF3T) as focal behavior and CIS-P categories [Direct Collaborative Processes on Negotiation Tasks/Goals (DCP1) and Affects (DCP2); Indirect Collaborative Processes on Facts (ICP1), Affects (ICP2), and Meaning (ICP3); Direct Rupture Markers on Relationship (DRM2); Indirect Rupture Markers as Linguistic Avoidance (IRM1) Affective Avoidance (IRM2), Self-esteem Regulation Strategies (IRM3), Indirect Allusions (IRM4), and Acquiescence (IRM5)] as conditional behaviors, and for the group of depressed patients **(B)**, considering the structural form Assertion (SF2P) as focal behavior and CIS-T categories [Direct Collaborative Interventions on Task/Goal (DCI1), Affects (DCI2), and Meaning (DCI3); Indirect Therapist Interventions on Facts (ICI1), Affects (ICI2), and Meaning (ICI3); Rupture Interventions as Linguistic Avoidance (RI1) and Hostility (RI3)] as conditional behaviors. Under each map, the results of the polar coordinate analysis are presented. The significance level was fixed at ^*^p < 0.05.

**Figure 4:**
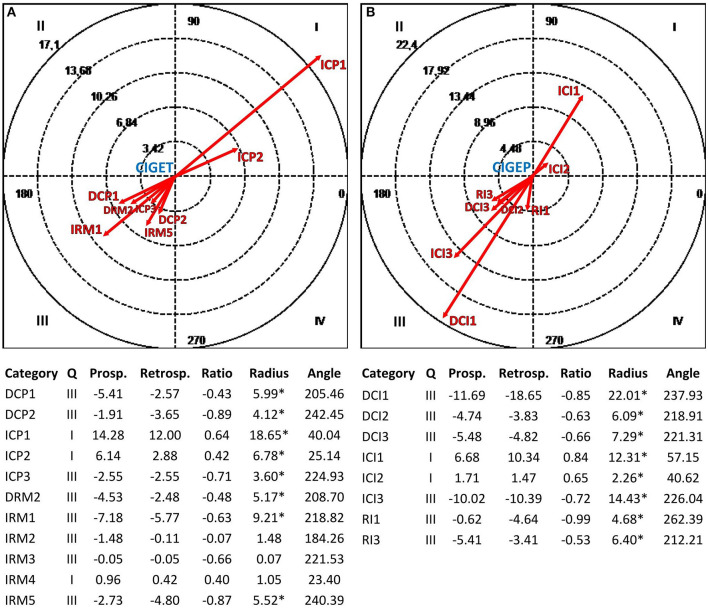
Vectorial maps of the statistically significant relationships for the therapist **(A)**, considering the communicative intent Global Exploration (CIGET) as focal behavior and CIS-P categories [Direct Collaborative Processes on Negotiation Tasks/Goals (DCP1) and Affects (DCP2); Indirect Collaborative Processes on Facts (ICP1), Affects (ICP2), and Meaning (ICP3); Direct Rupture Markers on Relationship (DRM2); Indirect Rupture Markers as Linguistic Avoidance (IRM1) Affective Avoidance (IRM2), Self-esteem Regulation Strategies (IRM3), Indirect Allusions (IRM4), and Acquiescence (IRM5)] as conditional behaviors, and for the group of depressed patients **(B)**, considering the communicative intent Global Exploration (CIGEP) as focal behavior and CIS-T categories [Direct Collaborative Interventions on Task/Goal (DCI1), Affects (DCI2), and Meaning (DCI3); Indirect Therapist Interventions on Facts (ICI1), Affects (ICI2), and Meaning (ICI3); Rupture Interventions as Linguistic Avoidance (RI1) and Hostility (RI3)] as conditional behaviors. Under each map, polar coordinate analysis results are presented. The significance level was fixed at ^*^p < 0.05.

**Figure 5:**
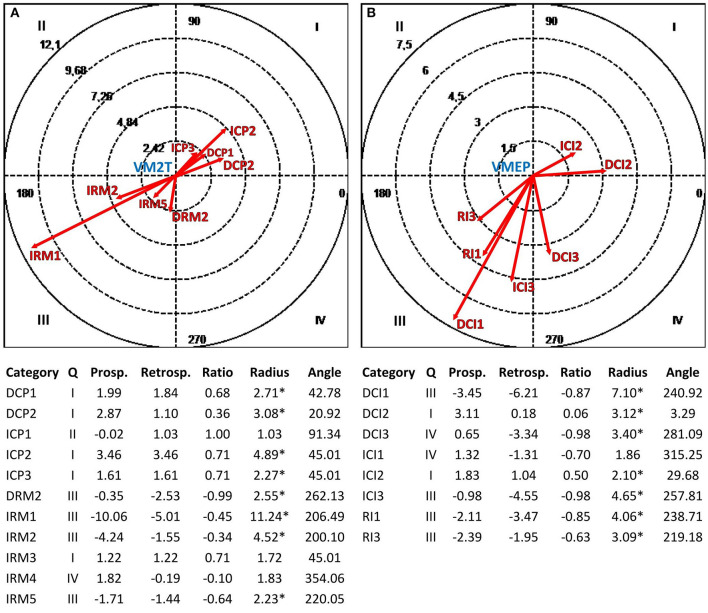
Vectorial maps of the statistically significant relationships for the therapist **(A)**, considering the vocal mode Connected (VM2T) as focal behavior and CIS-P categories [Direct Collaborative Processes on Negotiation Tasks/Goals (DCP1) and Affects (DCP2); Indirect Collaborative Processes on Facts (ICP1), Affects (ICP2), and Meaning (ICP3); Direct Rupture Markers on Relationship (DRM2); Indirect Rupture Markers as Linguistic Avoidance (IRM1) Affective Avoidance (IRM2), Self-esteem Regulation Strategies (IRM3), Indirect Allusions (IRM4), and Acquiescence (IRM5)] as conditional behaviors, and for the group of depressed patients **(B)**, considering the vocal mode Emotional (VMEP) as focal behavior and CIS-T categories [Direct Collaborative Interventions on Task/Goal (DCI1), Affects (DCI2), and Meaning (DCI3); Indirect Therapist Interventions on Facts (ICI1), Affects (ICI2), and Meaning (ICI3); Rupture Interventions as Linguistic Avoidance (RI1) and Hostility (RI3)] as conditional behaviors. Under each map, the results of the polar coordinate analysis are presented. The significance level was fixed at ^*^p < 0.05.

**Figure 6:**
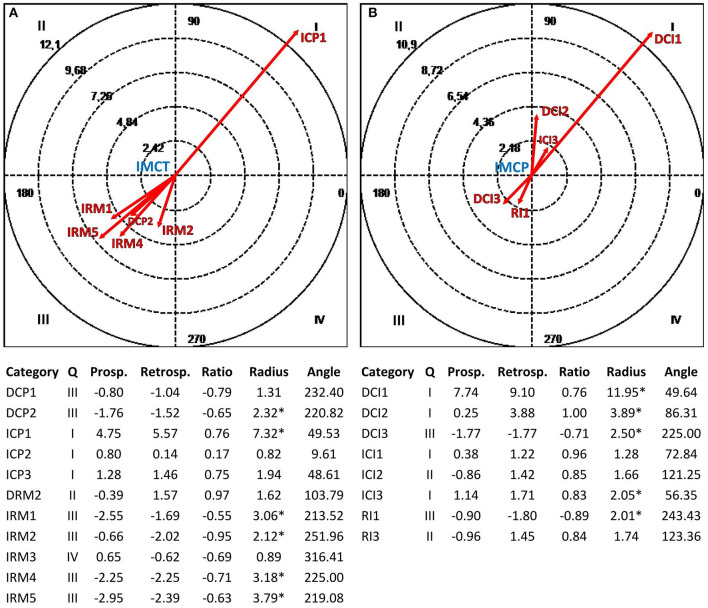
Vectorial maps of the statistically significant relationships for the therapist **(A)**, considering the interruption mode Cooperative (IMCT) as focal behavior and CIS-P categories [Direct Collaborative Processes on Negotiation Tasks/Goals (DCP1) and Affects (DCP2); Indirect Collaborative Processes on Facts (ICP1), Affects (ICP2), and Meaning (ICP3); Direct Rupture Markers on Relationship (DRM2); Indirect Rupture Markers as Linguistic Avoidance (IRM1) Affective Avoidance (IRM2), Self-esteem Regulation Strategies (IRM3), Indirect Allusions (IRM4), and Acquiescence (IRM5)] as conditional behaviors, and for the group of depressed patients **(B)**, considering the interruption mode Cooperative (IMCP) as focal behavior and CIS-T categories [Direct Collaborative Interventions on Task/Goal (DCI1), Affects (DCI2), and Meaning (DCI3); Indirect Therapist Interventions on Facts (ICI1), Affects (ICI2), and Meaning (ICI3); Rupture Interventions as Linguistic Avoidance (RI1) and Hostility (RI3)] as conditional behaviors. Under each map, the results of the polar coordinate analysis are presented. The significance level was fixed at ^*^p < 0.05.

Figures 3–**6** show the results of the polar coordinate analysis for the therapist and depressed patients. Each vectorial map represents the statistically significant associations between each communicative mode (i.e., each focal behavior detected from the reviewed literature) and the behaviors connected to the TA construction (i.e., conditional behaviors). The statistically significant association is shown both qualitatively (Quadrant I, II, III, or IV) and quantitatively (vector length). Again, the results are structured into sections based on the four verbal and non-verbal dimensions that we analyzed for the therapist and depressed patients. We will discuss the vectors with a length >1.96 (*p* < 0.05), expressing the relationships between focal behaviors' and conditional behaviors' activations in each vectorial map.

#### Relationships Between the Structural Forms Used by the Therapist and Depressed Patients and the Reciprocal Construction of the Therapeutic Alliance

Figure 3A shows the mutual activation (Quadrant I) between the structural form Question (SF3T) used by the therapist and the collaborative processes of depressed patients related to the TA construction. In particular, we can notice a strong mutual excitatory relationship with collaborative processes related to the events experienced by such patients (ICP1). Moreover, although with less intensity, there are mutually excitatory relationships with depressed patients' collaborative processes on feelings and/or thoughts related to their experiences (ICP2) and on the therapy goals and tasks (DCP1). On the other hand, in Figure 3B, there is a mutual activation (Quadrant I) between the structural form Assertion (SF2P) used by depressed patients and the therapist's collaborative interventions on the events experienced by this last one (ICI1).

#### Relationships Between the Communicative Intents Used by the Therapist and Depressed Patients and the Reciprocal Construction of the Therapeutic Alliance

In Figure 4A, there is above all a mutually excitatory relationship (Quadrant I) between the communicative intent Global Exploration (CIGET) used by the therapist and collaborative processes of depressed patients related to the events experienced (ICP1). Furthermore, there are mutual excitatory relationships with depressed patients' collaborative processes on feelings and/or thoughts related to their experiences (ICP2). Symmetrically, in Figure 4B, the depressed patients' use of the communicative intent Global Exploration (CIGEP) involves a mutual activation (Quadrant I) with the therapist's collaborative interventions on the events experienced by depressed patients (ICI1), and with less intensity, with collaborative interventions focused on thoughts and/or feelings about their experiences (ICI2).

#### Relationships Between the Vocal Modes Used by the Therapist and Depressed Patients and the Reciprocal Construction of the Therapeutic Alliance

In Figure 5A, the therapist's use of the vocal mode Connected (VM2T) determines mutually excitatory relationships (Quadrant I) with depressed patients' collaborative processes on feelings and/or thoughts related to their experiences (ICP2), feelings toward the therapist and therapy (DCP2), the therapy goals and tasks (DCP1), and the deep meaning of the events experienced (ICP3). On the other hand, in Figure 5B, the depressed patients' use of the vocal mode Emotional (VMEP) involves mutual activations (Quadrant I) with the therapist's collaborative interventions on patients' feelings toward the therapy and/or the therapist (DCI2) and on the feelings and/or thoughts of patients about their experiences (ICI2). Moreover, the vocal mode Emotional (VMEP) activates (Quadrant IV) the therapist's collaborative interventions on the meaning of the episodes that occur with patients during the psychotherapy session to identify behavioral patterns in the relationship with them (DCI3).

#### Relationships Between the Interruption Modes Used by the Therapist and Depressed Patients and the Reciprocal Construction of the Therapeutic Alliance

Figure 6A shows the mutually excitatory relationship (Quadrant I) between the therapist's use of the interruption mode Cooperative (IMCT) and depressed patients' collaborative processes related to the events experienced (ICP1). In Figure 6B, there are mutual activations (Quadrant I) between the depressed patients' use of the interruption mode Cooperative (IMCP) and therapist's collaborative interventions focused on the therapy goals and tasks (DCI1), the patients' feelings toward the therapy and/or the therapist (DCI2), and the meaning of patients' experiences (ICI3).

## Discussion

Our study aimed to analyze how specific verbal and non-verbal modes, implemented by the therapist and depressed patients, could influence and foster the reciprocal construction of a good TA, a relational and collaborative dimension that proved to be an active agent in the process of psychotherapy change (Colli and Lingiardi, 2009) during the mutual regulation processes emerging in the initial stages of therapy.

The findings presented propose a perspective of investigation on the psychotherapeutic exchange that emphasizes the importance of the joint action of *what* is said and *how* it is said, as an interacting system of verbal and non-verbal behaviors that acts by spreading information within a mutual regulation process between participants (Del Giacco et al., 2019). This notion of communication allows analyzing the therapeutic interaction by identifying those actions whereby both the therapist and the depressed patient participate in the TA construction and the verbal and non-verbal coordination processes. These aspects are at the basis of therapeutic change, as new ways for the patient to give meaning, interpret, and represent the inner reality and the surrounding world (Arístegui et al., 2004; Valdés and Krause, 2015). The results of the early TA study during the mutual regulation processes corroborate that the verbal and non-verbal behaviors of the therapist and depressed patients (who show difficulties in establishing and maintaining the TA because of their symptomatic characteristics) play a significant role in fostering collaborative behaviors that consolidate the therapeutic relationship in the initial stages of psychotherapy. All this confirms that the early TA lays the foundations for therapeutic change (Colli and Lingiardi, 2009; Ardito and Rabellino, 2011).

Concerning Verbal Mode-Structural Forms, the results confirm our hypothesis and corroborate the findings of Krause et al. (2016), according to which the therapist's structural form Question and the depressed patients' structural form Assertion foster the coordination between participants through collaborative behaviors. First of all, as in the study of Krause et al. (2016), we can notice that the therapist tends to ask more than depressed patients, while the latter tend to assert more than the former during the processes of TA building in the initial stages of psychotherapy. Of course, Verbal Mode-Structural Forms represent a surface characteristic of the communicative exchange between the therapist and patient; however, this result may provide information about the heterogeneity of the therapeutic process over time. According to Krause et al. (2016), these differences in using structural forms show the relational asymmetry between the therapist and patients where the roles are complementary: questions about the problems of the patient characterize the therapist's role, while assertions about their inner reality characterize patients. Moreover, this asymmetry is consistent with the idea of the initial phase of therapy as a moment of co-construction of the relationship and development of intersubjectivity, in which participants regulate each other according to the different verbal behaviors associated with their roles (Beebe et al., 2005). The studies of Krause et al. (2016) and Long (2001) emphasize that this asymmetry is reduced during the final stage of psychotherapy as if the former was preparatory to the latter. During this stage, indeed, both participants tend to affirm; moreover, the therapist performs actions aimed at making patients more responsible about the problem and its recovery to prepare them for the end of the therapy.

Our analyses show that the therapist's use of questions involves stable patterns and significant associations with collaborative processes by depressed patients, mainly related to the exploration of their experiences, emotions, and the goals of psychotherapy. Symmetrically, the depressed patients' use of assertions involves stable patterns and significant associations with collaborative interventions by the therapist on their experiences. Therefore, during the initial stages of psychotherapy, both questions of the therapist and assertions of depressed patients generate, together with the collaborative behaviors of the other, two self-sustaining systems that consolidate the therapeutic relationship within a mutual coordination process (Beebe, 2006). These behaviors are mainly at an experiential level for both participants and do not deepen the meaning of the internal representations of patients. Nevertheless, the use of questions stimulates depressed patients to give the therapist access to their emotional states related to these experiences and participate in the definition of therapeutic work. All this is consistent with the initial stage of psychotherapy when the therapist and patients are focused on laying the foundations of the therapeutic relationship (Safran and Muran, 2003). Thus, in clinical practice, the use of questions and assertions in the first stages of psychotherapy may promote collaborative behaviors that support the development and consolidation of a positive therapeutic relationship. Questions assume the function of a negotiating tool available to the therapist for the subsequent construction of new meanings. On the other hand, assertions become the expression of oneself and one's inner reality by depressed patients on which the therapist may act through his/her interventions for the construction of “new certainties” (Krause et al., 2016). We can conclude that questions and assertions, as regulatory strategies fostering the construction of a collaborative relationship, lay the foundations on which the therapeutic change rests and support its understanding.

Regarding Verbal Mode-Communicative Intents, the results confirm what we expected and are consistent with the findings of Dagnino et al. (2012), which underline that the therapist's and patients' intents of exploring (in our case the macro-category Global Exploration) affect the reciprocal coordination between participants through collaborative behaviors. As in the study of Dagnino et al. (2012), during the processes of building the TA, depressed patients use more global exploration (exploring, deepening, and focusing) than the therapist in the initial stages of psychotherapy. All this is consistent with the idea that the psychotherapy process requires an initial stage of inquiry and information exchange mainly focused on the exploration by patients (Dagnino et al., 2012).

As we can notice, the therapist's and depressed patients' global explorations involve similar stable patterns and significant associations with the reciprocal collaborative behaviors of participants, focused on the events experienced by patients and their feelings about these experiences. The communicative intents of exploring, deepening, and focusing -which constitute the global exploration- show the complementary nature of verbal interactions and collaborative behaviors of participants, implemented through circular schemes that foster the coordination processes and the TA construction (Heatherington, 1988; Dagnino et al., 2012). All this allows the construction of a relational space that promotes collaborative behaviors aimed at the joint work of the therapist and the depressed patient on the problems of the latter who, however, is the primary agent for subjective change (Reyes et al., 2008; Dagnino et al., 2012). As Valdés et al. (2005) pointed out, these exploratory intents lay the foundations for the subsequent processes of resignification and therapeutic change. The collaborative behaviors related to experiences and emotions emerging in the initial stages of therapy could be considered as necessary precursors “to raise awareness of better cognitive or affective adaptive patterns” (Valdés and Krause, 2015, p. 115) and to encourage cognitive and behavioral changes in the subsequent phases of building new meanings (Goldman et al., 2005). In clinical practice, these results may provide the therapist with empirical support to develop and consolidate an appropriate collaborative relationship at the basis of resignification processes, where there is a mutual communicative and emotional adaptation between participants: this is possible by performing interventions aimed at self-and mutual regulation through the speech and by encouraging the depressed patient to explore.

Concerning Vocal Modes, the results confirm our hypothesis and support the findings of Tomicic et al. (2015b) where the therapist's vocal mode Connected and the patients' vocal mode Emotional play a significant role in the coordination processes between participants at the basis of the TA construction and psychotherapy change. In our study, it emerged that depressed patients show a greater elaborative and emotional vocal mode than the therapist during the coordination processes. Compared with the study of Tomicic et al. (2015b), where the latter expressed a more elaborative vocal quality than the former, our result could be interpreted as the effect of psychodynamic psychotherapy. Especially in the early stages, indeed, this approach stimulates depressed patients to connect with their inner world and to define the unresolved problems and unconscious feelings, creating a space of intervention that the therapist may access to work on them (Busch et al., 2007; Gabbard, 2018).

Nevertheless, our analyses show that the therapist's use of an elaborative vocal mode involves stable patterns and significant associations with depressed patients' collaborative processes on feelings related to their experiences and the therapy as well as on the therapy goals and the meaning of the events experienced. According to Tomicic and Martínez (2011), during the psychotherapeutic process, the occurrence of vocal modes is heterogeneous and assumes a U-shape where the elaborative vocal mode characterizes the initial stages. Considering voice as a tool for transmitting psychological meanings and emotional states among participants (Tomicic et al., 2011), this vocal mode of the therapist promotes the development of the inter-mental space (Martinez Guzman et al., 2014) that receives patients and stimulates the latter to implement collaborative behaviors focused on reworking their emotional states and inner representations. At the same time, this inter-mental space supports intersubjective processes in depressed patients, encouraging their contribution to define and consolidate the relationship and therapeutic work with the therapist through continuous circular processes (Wiseman and Rice, 1989). Similarly, from the depressed patients' use of emotional vocal mode, there are stable patterns and significant associations with the therapist's collaborative interventions on patients' feelings related to the therapy and their experiences and on the meaning of episodes occurring during a psychotherapy session. The emotional vocal mode, characterizing the whole therapeutic process (Tomicic and Martínez, 2011), affects the emotional climate of sessions and the development of TA (Bauer et al., 2010). Voice reflects the speaker's emotional state that “allows the listener an empathetic understanding of the speaker him/herself” (Tomicic et al., 2009, p. 36). Therefore, vocal expression of emotions by depressed patients stimulates the therapist to consolidate the affective syntony that emerges in the psychotherapeutic relationship and to rework the emotional experience of patients through circular and continuous patterns (Beebe, 2006; Orsucci et al., 2016). At the same time, this vocal mode expresses the depressed patients' openness to their inner states, encouraging the therapist to implement interventions aimed at identifying dysfunctional patterns. Thus, in clinical practice, elaborative and emotional vocal modes, intertwining with the verbal dimension of the therapeutic dialogue (Jones and LeBaron, 2002), may become psychotherapeutic tools that support the therapist in self-and mutual regulation processes with depressed patients (Tomicic et al., 2009), increasing the effectiveness of interventions to consolidate the therapeutic relationships and the deepest reworking processes that prepare for change.

Regarding Interruption Modes, the results confirm our hypothesis and, in agreement with Li et al. (2005), show that cooperative interruptions activate coordination processes between participants through circular schemes (Beebe, 2006), assuming a mediating role in the TA construction and, consequently, in psychotherapy change^1^. As in the study of Oka et al.^1^, during the TA construction, the therapist implements more cooperative interruptions than depressed patients in the initial stages of psychotherapy. Within the therapeutic encounter, the relational asymmetry between patient and therapist implies that the latter is the one who has control of the conversational process (Fisher, 1984). Patients who ask for help recognize the therapist's position as an expert to rely on; the latter, therefore, has the professional power whereby he/she can interrupt to address the problems that the patient brings into the session (Stratford, 1998). Thus, the therapist's interruptions may assume collaborative potential when experienced by patients as “appropriate use of their expertise, to helpfully alter the direction or content of the therapeutic conversation” (Stratford, 1998, p. 388).

From our results, we can notice that the therapist's use of cooperative interruptions leads to stable patterns and significant associations with depressed patients' collaborative behaviors related to the events experienced. As we mentioned above, the initial stage of psychodynamic therapy represents a moment of acceptance and definition of the patient's problems in which the therapist guides the inquiry and, at the same time, leaves freedom of exploration to the former (Busch et al., 2007; Gabbard, 2018). During the therapeutic dialogue, the therapist invades the elaborative space of depressed patients with the intent of agreeing, supporting, and clarifying, that is implementing interruptions that, according to Ng et al. (1995) and Stratford (1998), promote the patients' exploratory behaviors and create an inter-mental space where participants develop and consolidate the therapeutic relationship (Martinez Guzman et al., 2014). On the other hand, from the depressed patients' use of cooperative interruptions, there are stable patterns and significant associations with the therapist's collaborative interventions on the therapy goals, patients' feelings related to the therapeutic relationship, and the meaning of their experiences. This result shows that, during the TA construction in the initial stages of therapy, depressed patients cooperatively interrupt to express involvement and participation in the therapeutic dialogue (Tannen, 1994; Cafaro et al., 2016), activating intersubjective processes that feed the inter-mental space with the therapist through continuous circular processes (Beebe, 2006; Martinez Guzman et al., 2014). This context allows the latter to implement collaborative interventions aimed, on the one hand, at consolidating the therapeutic relationship and work and, on the other hand, at promoting the redefinition of depressed patients' representations (Goldberg, 1990). In clinical practice, during the initial stages of psychotherapy, cooperative interruptions enrich the meaning and strength of the speech: they could be facilitators for the therapist and indicators of the depressed patients' involvement level. Therefore, the therapist may use these interruptions both to encourage the exploratory processes with the depressed patient and to orient the mutual coordination processes at the basis of the TA construction and psychotherapy change.

In support of our results and by way of example, the two following clinical vignettes (Table 9) show possible combinations of communicative behaviors for a good and a poor TA, respectively.

**Table 9:** Clinical vignettes.

**Turn**	**Role**	**Transcription**	**VeM-SF**	**VeM-CI**	**VoM**	**IM**	**CIS-T**	**CIS-P**
**Clinical vignette 1**								
180	T	How do you feel about talking about stuff like that again?	Question	Global Exploration	Connected	/	DCI on Affects	
181	P	It's strange… I'm not used to talking about my things, but I feel calm because it was something I wanted to do for me.	Assertion	Global Exploration	Emotional	/		DCP on Affects
182	T	Calm how? //(<2”)	Question	Global Exploration	/	/	DCI on Affects	
183	P	//Well, you know, it's hard to have a dialogue with my mom without a figh-//	Assertion	Global Exploration	Emotional	Cooperative		ICP on Facts
184	T	//Do you feel anger growing with her too?	Question	Global Exploration	Connected	Cooperative	ICI on Affects	
185	P	Yes…I try to tell her what I have inside, but she doesn't listen to me and stays firm in her beliefs…so I start shouting…	Assertion	Global Exploration	Emotional	/		ICP on Facts
**Clinical vignette 2**								
106	T	It seems to me that you're behaving with your boyfriend the same way as you are with your fathe-//	Assertion	Resignifying	Declarative	/	ICI on Meaning	
107	P	//No, it's not like you're sayin-//	Denial	Global Exploration	Declarative	Intrusive		DRM on Relationship
108	T	//but, when you stop to put together the relationship you have with your boyfriend and that one with your father, you don't seem so sure anymore-//	Assertion	Resignifying	Declarative	Intrusive	RI of Hostility	
109	P	//My father was a person who disappeared for days, but you know how fathers are… they're always busy at work.	Assertion	Global Exploration	Declarative	Intrusive		IRM of Affective Avoidance

*T, Therapist; P, Patient; VeM-SF, Verbal Mode-Structural Form; VeM-CI, Verbal Mode-Communicative Intent; VoM, Vocal Mode; IM, Interruption Mode; CIS-T, Collaborative Interactions Scale-Therapist; CIS-P, Collaborative Interactions Scale-Patient; DCI, Direct Collaborative Intervention, ICI, Indirect Collaborative Intervention; RI, Rupture Intervention; DCP, Direct Collaborative Process; ICP, Indirect Collaborative Process; DRM, Direct Ruptures Marker; IRM, Indirect Rupture Marker; /, indicates the not-coded behaviors; //, indicates the speaking turn interruption; (<2”), indicates a speech <2 s in duration*.

Clinical vignette 1 emphasizes what emerged so far and how the interaction of verbal and non-verbal communicative modes, analyzed in our study and implemented by the therapist and depressed patient, leads to the building of a good alliance and the consolidation of the therapeutic relationship. Clinical vignette 2, on the contrary, shows the series of communicative exchanges bringing to the rupture of TA due to the combination of some verbal and non-verbal modes by the therapist and patient that, according to the literature (Li et al., 2005; Dagnino et al., 2012; Tomicic et al., 2015b; Krause et al., 2016,^1^), may negatively influence the processes of change and relational construction. In turn 106, the therapist tries to resignify the patient's experience by affirming with conviction a particular state of reality. However, the patient reacts by intrusively interrupting and denies with certainty by attacking the relationship with the therapist (turn 107). In turn, the therapist intrusively interrupts through a new resignification that affirms with conviction and hostility (turn 108). The patient replies by interrupting again in an intrusive way and affirms with conviction his inner reality by isolating affection (turn 109). It should be noted that, despite the patient's communicative intent of global exploration, the presence of declarative and intrusive modes intertwining with the verbal component affects the meaning of the speech emitted, hindering the process of change and bringing to the rupture of TA. Probably, since these are the initial stages of the therapy (the first three sessions), the attempt of resignification that the therapist affirms with conviction is too premature to be supported by the depressed patient, generating an escalation of conflictual ruptures between participants that deteriorate the TA.

The results obtained advance in understanding the verbal and non-verbal communication modes that foster the TA construction between therapist and depressed patients in the initial stages of psychodynamic psychotherapy. Precisely, the study provides a measure of those elements of communication that may sustain depressed patients to overcome the difficulties in accessing their inner world and emotions and in regulating their relational distance in interaction with the therapist (Valdés, 2014; Valdés and Krause, 2015). These represent typical aspects of the functioning profile of depressed patients that derive from the first cognitive-affective representations and impact on the development and maintenance of the TA (Levy and Wasserman, 2009; Balsters et al., 2012; Smirnova et al., 2018). We believe, therefore, that these results, on the one hand, may consolidate knowledge on verbal dynamics and, on the other hand, may reveal aspects unexplored in the Italian context on vocal and interruption modes that, together with the former, may guide interventions with this kind of patients to increase the therapeutic effectiveness and lay the foundations for change.

The observational methodology application, both through the integrative procedure of an *ad hoc* indirect observation instrument and an observation tool with deductive (or theoretical) categories and through the use of quantitative statistical analysis techniques, has proved effective in obtaining relevant information on the dynamics existing between patient and therapist. In particular, the complementary use of lag sequential analysis and polar coordinate analysis allows a rigorous, objective, and exhaustive evaluation of the reality of the therapeutic exchange (Anguera et al., 2018). In our study, these analyses were performed considering 10 retrospective lags (from lag-10 to lag-1) and 10 prospective lags (from lag+1 to lag+10), unlike the usual practice of including only five lags (Sackett, 1980). Given the type of subject, the purpose of the study, and the characteristics of participants, we made this choice to obtain a greater wealth of information from the complexity of the interactive dynamics between therapist and depressed patients. The mixed methods approach, which includes this methodology, has allowed observing the ecological context of the therapeutic exchange through objective measures increasing the knowledge on the processes related to the TA construction (Creswell and Plano Clark, 2017; Anguera et al., 2018).

However, this study is not exempt from limitations. The first one is related to the theoretical approach of psychotherapy. Our research only considered psychodynamic psychotherapy but, as a future objective, it would be interesting to extend the study of the dynamics between communication and TA building to other types of psychotherapeutic approaches (e.g., cognitive-behavioral therapy, systemic therapy) to investigate the potential precursors of change in each of them. Second, we only contemplated therapies conducted by the same female therapist; for future developments, it would be useful to include the study of psychotherapies with male therapists to assess the presence of gender differences in the indicators underlying the change. Third, we only analyzed the first three sessions of each psychotherapy, but it would be useful to extend the study to complete therapies to understand how the communicative modes influence the whole process and the psychotherapy outcome (e.g., by performing pre-post treatment studies), connected to change. Fourth, we observed 20 psychotherapy sessions (equivalent to 6,237 speaking turns); although it is an adequate number to collect a large amount of data and to detect hidden structures between constructs from the investigative perspective of the observational method (Anguera et al., 2017), it corresponds to the material produced by only seven patients from a clinical perspective. It would be useful to progressively increase the number of participants to extend the research and carry out further investigations such as the *multiple case study analysis* that allows detecting regularities between cases that are similar in some ways and homogeneous in the selection criteria (Stake, 2006). Fifth, our study focused on the interaction between communication and TA in patients with depressive symptoms. It could be interesting to extend the research to other types of disorders (e.g., anxiety, eating disorders, affective dysregulation) to trace behavioral patterns and significant associations related to change that are specific to each of them. Sixth, we focused on communication modes that have a positive impact on building a collaborative relationship between patient and therapist. However, it would be useful to extend the research by evaluating those indicators that may have a negative impact or hinder therapeutic change. Finally, our study took into account the processes of mutual regulation between therapist and patient; however, it would be useful to deepen the self-regulatory processes to understand how they affect the internal organization of each participant during the construction of change.

## Data Availability Statement

The datasets generated for this study are available on request to the corresponding author.

## Ethics Statement

The Psychology Interdepartmental Ethics Committee of the University of Padua (Italy) evaluated and approved this investigation. The study has been conducted following the ethical guidelines and procedures of the Interdepartmental Laboratories for Research and Applied Psychology (L.I.RI.P.A.C.), to which the DPS belongs, based on Italian law no. 196/03 about privacy and confidentiality and the ethical standards for research established by the American Psychological Association (2017). All participants gave their written informed consent to participate in the research in conformity with the Declaration of Helsinki before making the audio recording and data collection; the study was conducted after the end of psychotherapies. Personal information of participants was replaced and not provided to the coders of audio recordings and transcripts to guarantee confidentiality.

## Author Contributions

LD documented, designed, drafted, and wrote the manuscript. Moreover, he trained and supervised the coders and accomplished statistical analyses. SS supervised the sample recruitment, while MA supervised the method and procedure sessions and statistical analyses. SS and MA revised the manuscript for theoretical and intellectual content. Finally, all authors provided the final approval of the version to be published.

### Conflict of Interest

The authors declare that the research was conducted in the absence of any commercial or financial relationships that could be construed as a potential conflict of interest.

## Footnotes

^1^Oka, M., Tambling, R. B., Walker, J., Seedall, R. B., Robinson, W. D., and Anderson, S. R. (in press). Therapist interruptions: an examination of gender and therapeutic alliance in couple therapy. *J. Mar. Fam. Ther*.

^2^The constructs of the cited studies (*asking, asserting*, and *exploring* for the verbal dimension; *elaborating, expressing emotions*, and *cooperatively interrupting* for the non-verbal dimension) will correspond to the variables operationalized in the observational instrument that analyzes verbal and non-verbal communication in this research.
