# Erratum: The Action of Verbal and Non-verbal Communication in the Therapeutic Alliance Construction: A Mixed Methods Approach to Assess the Initial Interactions With Depressed Patients

**DOI:** 10.3389/fpsyg.2020.01351

**Published:** 2020-05-27

**Authors:** Frontiers Production Office

**Affiliations:** Frontiers Media SA, Lausanne, Switzerland

**Keywords:** verbal and non-verbal communication, performative language, therapeutic alliance construction, mutual regulation, coordination processes, psychotherapy process, depression, mixed-methods approach

Due to an error in the typesetting process, the references to footnote one in the published article were incorrect. The in-text citation “Oka et al.” was omitted and the superscript 1, linking to the corresponding footnote, was misplaced.

The publisher apologizes for this mistake. The original article has been updated.

