# Supplementary material for: The Action of Verbal and Non-verbal Communication in the Therapeutic Alliance Construction: A Mixed Methods Approach to Assess the Initial Interactions With Depressed Patients
Source: Front Psychol. 2020 Feb 21;11:234. doi: 10.3389/fpsyg.2020.00234 (PMC7047748; doi:10.3389/fpsyg.2020.00234)
Supplement: Supplementary file 1 [file Table_1.PDF]

Supplementary Material

Supplementary Appendix I. Description of the CMASP dimensions and categories (retrieved from Del Giacco et al., 2019)

Dimension	Categories	Description	Code
Verbal Mode-Structural Form (VeM-SF)<br><br>It concerns the formal structure of the speech by which the speaker expresses the verbal mode (it corresponds to the propositional component of the speaker's speech).	Courtesies	The speaker's speech is in the form of terms expressing receptiveness to the communication according to social conventions (e.g., *"Good morning"*, *"Goodbye"*, *"Thank you", "Your welcome"*).	SF1
	Assertion	The speaker's speech expresses something he/she considers true, or it refers to a specific state of things (e.g., *"I feel empty"*, *"I can hardly concentrate"*).	SF2
	Question	The speaker's speech is in the form of a request for specific information (e.g., "*Would you like to tell me the problem?"*, *"And this laziness hum for example in what…"*, *"So, you're not Italian…"*).	SF3
	Agreement	The speaker's speech recognizes the truth of the other's statement (e.g., *"Mm-hm"*, *"Right"*, *"Yes"*, *"Of course"*, *"Perhaps"*, *"All right"*).	SF4
	Denial	The speaker's speech refuses to recognize (or rejects) the truth of something said by the other (e.g., *"No"*, *"In no way"*, *"Absolutely no"*).	SF5
	Direction	The speaker's speech encourages the listener towards cognitive, emotional, or behavioral actions by guiding the other's behavior (e.g., *"Tell me what's wrong"*).	SF6
Verbal Mode-Communicative Intent (VeM-CI)<br><br>It concerns the underlying intention of the speaker's speech (it corresponds to the performative	Acknowledging	The speaker's communicative intent is to take the other's point of view about the other's experience while presuming no knowledge of it (presuming knowledge of the speaker's experience only), (e.g., *"Mm-hm"*, *"Ok"*; *"Exactly"*, *"Mh"*, *"Right"*, *"Good morning"*, *"Goodbye"*, *"Thank you"*, *"Your welcome"*).	CI1
	Informing	The speaker's communicative intent is to supply (or request for), information about the here and now of psychotherapy in the form of data, facts, resources, theory, and assessment parameters. The information may	CI2

component of communication).		be specifically related to the counseling process, therapist behavior, or arrangements (time, place, fee, and so on), (e.g., T: *"We'll meet once again, and then we'll take stock of the situation"*).	
	Exploring	The speaker's communicative intent is to ask for information about knowledge, events, feelings, or the causes of content or behavior. (e.g., T: *"Would you like to tell the reason you are here?*"; T: *"How was your move to Padua?"*). Moreover, the speaker may provide the information required by the other focusing on knowledge, events or feelings (e.g., P: *"My parents are divorced"*); he/she may talk about new contents in the form of stories as well as descriptions of past or present experiences (e.g., P: *"When I was a child, I liked to sleep with my parents"*); he/she may describe a feeling or emotional state (e.g., P: *"I've no energy and I always feel sad"*).	CI3
	Deepening	The speaker's communicative intent is to deepen the description, presentation, or discovery of some contents. The speaker may realize it: a) by verifying the truthfulness of an assertion made by the other which is questioned (e.g., P: *"I got so mad when he said to me those words, but you I'm fine on my own"* – T: *"So, don't you care of what the others say?"*); b) by correcting the comprehension of the other (e.g., T: *"If I've understood correctly, it sounds like your problem is due to relationships"* – P: *"No, the problem is only with my mother"*); c) by corroborating something stated (an opinion, facts, or new contents given or requested by the other) (e.g., T: *"So, you're one of the most aged"* – P: *"Yes, I was selected for my age"*); d) by requesting for information about the content of the other's communication (e.g., P: *"I've called him many times but…nothing"* – T: *"In other words, hasn't he called you back anymore?"*).	CI4
	Focusing	The speaker's communicative intent is to direct the attention and efforts towards a specific topic of conversation. The speaker may realize it: a) by introducing or addressing a topic (e.g., P: *"Well, I would like to start with the reasons"*); b) by returning to a topic (e.g., T: *"So, getting back to what we were talking about"*); c) by summarizing a content (e.g., T: *"Today we have spoken about many things"*); d) by defining the limits of	CI5

		a given content (e.g., T: *“I’d like to focus on the relationship with your boyfriend”*).	
	Temporizing	Speaker’s communicative intent is to assume a suspended position as regards the other’s communication. This intent allows the speaker to get in touch with his/her thoughts and feelings or, on the contrary, to avoid facing the requests of the previous speech, momentarily (e.g., T: *“How did you feel?...How..”* – P: *“How I felt…”*).	CI6
	Attuning	The speaker’s communicative intent is to understand or be understood by the other. He/she may realize it: a) by verifying his/her comprehension with a careful examination of what he/she understood about the other’s communication (e.g., T: *“Let me get this straight, you’re telling me your mom doesn’t know you smoke”*); b) by telling the other how his/her actions or thoughts are being understood (e.g., T: *“In other words, you think your mood is due to your parents’ divorce”*); c) by communicating to the other that his/her actions or thoughts are understood (e.g., T: *“Now I see, in other words, you’re a sophomore in University”*). Moreover, to express attuning, the speaker may harmonize with the other showing an emotional connection to his/her reality (e.g., T: *“I imagine it’s a difficult situation”*). Finally, the speaker may perform this communicative intent by providing feedback: a) to validate or discourage the other’s behaviors, meanings or feelings (e.g., T: *“Don’t worry, go on”*); b) to show the other’s affections or tell the emotional impact that the other had on the speaker (e.g., T: *“I’m making you angry”*).	CI7
	Resignifying	The speaker’s communicative intent is: a) to offer a new perspective on content (e.g., T: *“Maybe, there is also the fear of not being understood”*); b) to connect contents to one another (e.g., P: *“I realize that I tend to get angry at my boyfriend like my father”*); c) to recognize or establish a psychological working model (e.g., T: *“You have a very rigid way of facing things*); d) to question a content (e.g., T: *“Well, but it seems you’re afraid to understand you can do it on your own”*).	CI8
Vocal Mode (VoM)	Reporting	The listener has the impression of a detached speech emitted by the speaker like he/she is reporting, narrating, or exploring contents without any emotional involvement. The speaker’s voice seems to attribute a	VM1

It concerns the underlying intentions of the speaker's speech associated with both a peculiar combination of acoustic parameters (tone, intensity, duration, and timbre) and a specific way such a speech affects the listener of communicative exchange, regardless of the verbal content. The "emotional" categories building follows the principle of universal emotions recognition.		detached quality to speech, and emotional disconnection (and/or emotional distance) seems to characterize what is being said. A typical vocal parameter of this category is the repetitive prosody (concerning the tone) which, in turn, presents an agogic accent (concerning the tone) and high variation in the dynamic (concerning the intensity). Finally, the speech is usually characterized by fluid pace (concerning the duration).	
	Connected	The listener has the impression of an elaborative speech emitted by the speaker and oriented towards the other. The speaker's voice seems to attribute to speech the quality of being connected and/or attuned to oneself and the other, giving the latter the space to intervene. The distinctive vocal parameters of this category are the anti-cadence, characterizing the end of the sentence expressed, and agogic accent (concerning the tone) with a soft-vocal attack (concerning the duration). Finally, the pace may present pauses and loss of fluidity (e.g., extensions, repetitions and so on), concerning the duration.	VM2
	Declarative	The listener has the impression of a secure, instructive, engaged or convinced talk emitted by the speaker. The speaker's voice seems to attribute the quality of certainty and conviction to the speech like he/she is instructing (or explaining to) the other, or like he/she seems very sure of (engaged in) what he/she is saying. The other has a little space for intervening. The peculiar vocal parameters of this category are the suspended or anti-cadence, characterizing the end of the sentence expressed, and agogic and/or dynamic accent (concerning the tone) with a hard-vocal attack (concerning the intensity). The pace is usually fluid (concerning the duration) while the intensity may present an average volume increased (concerning the intensity).	VM3
	Introspective	The listener has the impression of an introverted speech emitted by the speaker. The speaker's voice seems to attribute to speech the quality of being directed towards oneself like he/she is connected with one's own internal world or in a dialog with oneself. The distinctive vocal parameters of this category are an average volume decreased and dynamics decrescendo (concerning the intensity). Sometimes, this vocal mode may present a reduced speed and long pauses (concerning the duration).	VM4

Emotional-Positive	The listener has the impression of a positive-emotional speech emitted by the speaker. The speaker's voice seems to attribute positive affection and/or positive emotional strength to speech. This vocal mode expresses the speaker's positive emotion (e.g., cheerfulness, happiness, sweetness, excitement, charm, understanding) modulating the verbal component of the speech (e.g., a laugh, shrill or sweet voice may accompany it) or, on the contrary, the effort to contain the emotion. A typical parameter of this category is the timbre, characterizing speech with variation in color and bright (Clear/Bright and Clear/Opaque), associated with changes in the sounding system (e.g., the shape that mouth assumes when someone smiles) expressing positive affection. Moreover, this category is often associated with a soft-vocal attack.	VM5
Emotional-Negative	The listener has the impression of a negative-emotional speech. The speaker's voice seems to attribute the quality of negative emotion and/or negative emotional strength to speech. This vocal mode expresses the speaker's negative emotion (e.g., anger, sadness, fear, tension) modulating the verbal aspect of the speech (e.g., sobbing, broken voice, trembling voice, snort may accompany it) or, on the contrary, the effort to contain the emotion. A typical parameter of this category is the timbre, characterizing speech with variation in color and bright (Clear/Bright, Dark/Bright, and Dark/Opaque), associated with changes in the sounding system (e.g., the nasal congestion when someone cries, or the tension of the vocal cord when someone is nervous) expressing negative affection. Finally, an increased volume and/or a non-fluid pace may characterize this vocal mode.	VM6
Pure Positive Emotion	The speaker's voice quality expresses a positive emotional state (e.g., doing a half-smile, laughing) without uttering any verbal content. The speaking turn is characterized only by vocalizations, due to changes in the sounding system, expressing a positive emotion and no utterance precedes or follows.	VM7
Pure Negative Emotion	The speaker's voice quality expresses a negative emotional state (e.g., crying, sighing) without uttering any verbal content. The speaking turn is characterized only by vocalizations, due to changes in the sounding	VM8

		system, expressing negative emotion and no utterance precedes or follows.	
Interruption Mode (IM)<br><br>It concerns the interrupter's behaviors, implemented to take the floor (successfully or not), for supporting or hindering the communicative flow of the current speaker.<br>These modes analyze both the potential violations of the transition relevance place (TRP) by the interrupter, the impact the interruption has on the other participant, and the "reaction" that this last one implements towards the interrupter. Each mode is defined by the time and manner in which the interrupter takes the floor and by the objective of the interruption.	Cooperative-Concurrence	This kind of interruption enables the interrupter to show agreement, validation, understanding, compliance, or support to the current speaker. Sometimes, the interruption also aims to extend or elaborate on the idea presented by the speaker.	IM1
	Cooperative-Assistance	This interruption mode enables the interrupter to sustain the current speaker by providing a word, phrase, sentence, or idea when the interrupter perceives the current speaker needs help.	IM2
	Cooperative-Clarification	The interrupter usually implements this kind of interruption mode to understand the message sent by the current speaker. The ultimate goal of the interruption is to make sure that the current speaker clarifies or explains a previously expressed piece of information the listener is dubious. In other words, when the listener is unclear about a piece of information the current speaker has just expressed, he/she interrupts this last one to request clarifications.	IM3
	Cooperative-Exclamation	The interrupter implements this mode to show rapport and coparticipant involvement by expressing surprise to the previous utterance of the speaker.	IM4
	Intrusive-Disagreement	The interrupter intervenes to show disagreement about what the speaker is saying and wants to correct or express his/her opinion immediately.	IM5
	Intrusive-Floor taking	The interrupter intervenes to develop the topic of the current speaker by taking over the floor from this last one. Generally, the interrupter does not intend to change the topic of the speaker, but only express his/her opinion, idea, thoughts, by taking the floor.	IM6
	Intrusive-Competition	This kind of interruption is characterized by a simultaneous speaking in which both participants interrupt each other to complete their speech, generating a real fight for the floor. In such an interruption, the one who first interrupted manages to take the floor and to prevent the other to end his/her speech.	IM7
	Intrusive-Topic change	The interrupter intervenes to change the topic by cutting the speech of the current speaker. The interrupter is somewhat more aggressive than in the	IM8

	floor-taking situation because he/she must accomplish the task of changing the topic.	
Intrusive-Tangentialization	In this kind of interruption, the listener interrupts to summarize the information sent by the current speaker, reflecting his/her awareness. In other words, since the interrupter does not want to listen to the same information repeatedly, then he/she intervenes to summarize one or more pieces of the previously expressed information; in this way, he/she minimizes the message sent by the current speaker. Tangentialization prevents the interrupter from listening to an unwanted piece of information because either the information has been presented previously or the listener through other channels already knows the information.	IM9
Neutral interruption	This kind of interruption mode is neither cooperative nor intrusive (it does not violate the principles of turn change). It occurs when the speaker pauses or stops the talk, creating uncertainty about his/her intention to continue the speech, and the interrupter takes the floor and starts to talk. The central aspect of this kind of interruption is that the speaker’s speech appears incomplete due to his/her stop.	IM10
Failed Interruption	A simultaneous speech characterizes the present interruption mode, but there is no turn exchange as in the IM Intrusive-Competition. It occurs when the listener tries to intervene interrupting, but he/she stops before finishing the intruding speech since the current speaker continues talking. This last one ignores the interrupter and continues talking until he/she finishes. In other cases, the interrupter stops before completing his/her intruding speech since he/she understands the speaker wants to continue talking.	IM11

References

Del Giacco, L., Salcuni, S., and Anguera, M. T. (2019). The Communicative Modes Analysis System in Psychotherapy From Mixed Methods Framework: Introducing a New Observation System for Classifying Verbal and Non-verbal Communication. *Front. Psychol*. 10:782. doi: 10.3389/fpsyg.2019.00782
