# Supplementary material for: The Action of Verbal and Non-verbal Communication in the Therapeutic Alliance Construction: A Mixed Methods Approach to Assess the Initial Interactions With Depressed Patients
Source: Front Psychol. 2020 Feb 21;11:234. doi: 10.3389/fpsyg.2020.00234 (PMC7047748; doi:10.3389/fpsyg.2020.00234)
Supplement: Supplementary file 2 [file Table_2.PDF]

Supplementary Material

Supplementary Appendix II. Scales, subscales, and categories of the Collaborative Interactions Scale-Revised (adapted from Colli et al., 2014)

Scale	Description	Code
CIS-Therapist (CIS-T)		
Form of Therapist Interventions (TI)		
Supportive	Interventions aiming to sustain the patient or reinforce his behavior/functioning.	TI1
Explicative	Interventions aiming to explain something to the patient.	TI2
Explorative	Interventions aiming to explore or stimulate the patient's elaboration.	TI3
Expressive	Interventions in which is predominant the interpretative stance by the therapist (e.g. interpretations, confrontations, and observations).	TI4
Direct Collaborative Interventions (DCI)		
Task/Goal	The therapist focuses on tasks/goals of therapy.	DCI1
Affects	The therapist focuses on desires and wishes toward the therapist and/or the therapy and/or focuses on feeling and/or thoughts, help patients in makes clear intensity or quality of his/her feelings or attitude toward the therapist or the therapy.	DCI2
Meaning	The therapist focuses on the meaning of an episode with the patient, connects an event with the patient to other issues or identify a pattern in the relationship with the patient.	DCI3
Meta Communication	The therapist talks about his experience in relationship with the patient in order to overcome an impasse, to repair an alliance rupture, or to improve the collaboration level.	DCI4

Indirect Therapist Interventions (ICI)		
Facts	The therapist focuses on significant facts and/or introduces topics or elements within a topic.	ICI1
Affects	The therapist focuses on feelings and/or thoughts, helps patients to make clear intensity or quality of his/her feelings or attitude.	ICI2
Meaning	The therapist talks about the meaning of events or connects a topic to a topic or to a schema, etc.	ICI3
Rupture Interventions (RI)		
Linguistic Avoidance	The therapist changes the offhand topic.	RI1
Affective Avoidance	Therapist talks in technical jargon or intellectualizes. Therapist interventions are not focused on patient's concrete experience.	RI2
Hostility	The therapist is hostile, sarcastic and/or seems to compete with the patient.	RI3
Perseveration	The therapist perseveres on a topic excessively. He is not tuned with the patient's answers.	RI4
Lack of Clarity	Therapist interventions are confused about the formal or meaning level. Interventions are not easy to understand.	RI5
CIS-Patient (CIS-P)		
Direct Collaborative Processes (DCP)		
Negotiation Tasks/Goals	The patient talks about tasks and/or goals of therapy in order to negotiate it with the therapist.	DCP1
Affects	Patients talk about her/his needs in relation to therapy and/or therapist and/or about his/her feeling, thoughts, makes clear intensity and/or quality of his/her feelings and/or attitude toward the therapist and/or the therapy.	DCP2
Meaning	The patient talks about the meaning of an event of the therapeutic relationship, and/or connects an episode with the therapist or other episodes outside therapy and/or to a schema.	DCP3
Indirect Collaborative Processes (ICP)		
Facts	The patient talks about new significant facts, introduces a topic or elements within a topic.	ICP1
Affects	The patient talks about his/her feeling and/or thoughts, makes clear intensity or quality of his/her feelings or attitude.	ICP2

Meaning	The patient talks about the meaning of events or connects a topic to a topic or to a schema, etc.	ICP3
Direct Rupture Markers (DRM)		
Task/Goal	The patient doesn't agree with the therapist about therapy tasks and/or goals.	DRM1
Relationship	The patient criticizes the therapist as a person and/or for his/her competence, feels uncomfortable with the therapeutic relationship.	DRM2
Discouragement	Patient doubts about feeling better and/or being in therapy. He complains about the lack of progress.	DRM3
Parameters	The patient complains about parameters of therapy (e.g., session time, fee).	DRM4
Indirect Rupture Markers (IRM)		
Linguistic Avoidance	The patient uses linguistic avoidance (talks in a wordy manner and/or spends an inordinate amount of time talking about other people and their doings and/or overly elaborates non-significant stories and so on, changes topic or tangentially answers to therapist intervention, short answers to therapist open question).	IRM1
Affective Avoidance	The patient makes use of emotional withdrawal strategies (denies evident affective state, intellectualizes about his/her inner experience).	IRM2
Self-esteem Regulation Strategies	The patient uses self- esteem regulation strategies (self-enhancing strategies and/or self-justifying statements and/or is self-critical or self-blaming).	IRM3
Indirect Allusions	The patient alludes to negative sentiments and/or concerns about the therapeutic relationship through a thematically linked discussion of out-of-session events or relationships.	IRM4
Acquiescence	Patient interacts in an acquiescent manner.	IRM5

Note. The authors granted permission to use the CIS-R scheme.

References

Colli, A., Gentile, D., Condino, V., and Lingiardi, V. (2014). *Collaborative Interactions Scale Revised (CIS-R). Coding Manual*. Rome, IT: Sapienza University.
